# Panel miRNAs are potential diagnostic markers for chronic kidney diseases: a systematic review and meta-analysis

**DOI:** 10.1186/s12882-024-03702-y

**Published:** 2024-08-13

**Authors:** Gantsetseg Garmaa, Rita Nagy, Tamás Kói, Uyen Nguyen Do To, Dorottya Gergő, Dénes Kleiner, Dezső Csupor, Péter Hegyi, Gábor Kökény

**Affiliations:** 1 Institute of Translational Medicine, Semmelweis University, Nagyvárad tér 4, Budapest, 1089 Hungary; 2 Center for Translational Medicine, Semmelweis University, 1085 Budapest, Üllői út 26, Budapest, Hungary; 3 Department of Pathology, School of Medicine, Mongolian National University of Medical Sciences, Ulan-Bator, 14210 Mongolia; 4 Heim Pál National Pediatric Institute, Üllői út 86, Budapest, 1089 Hungary; 5 Department of Stochastics, Institute of Mathematics, Budapest University of Technology and Economics, Budapest, Hungary; 6 András Pető Faculty, Semmelweis University, 1Üllői út 26, Budapest, 1089 Hungary; 7 Department of Pharmacognosy, Semmelweis University, Üllői út 26, Budapest, 1085 Hungary; 8 Department of Pharmacy Administration, University Pharmacy, Semmelweis University, Budapest, Hungary; 9 Institute for Translational Medicine, Medical School, University of Pécs, Szigeti út 12, Pécs, 7624 Hungary; 10 Institute of Clinical Pharmacy, University of Szeged, Szikra út 8, Szeged, 6725 Hungary; 11 Institute of Pancreatic Diseases, Semmelweis University, Tömő út 25-29, Budapest, Hungary; 12 International Nephrology Research and Training Center, Semmelweis University, Nagyvárad tér 4, Budapest, 1089 Hungary

**Keywords:** microRNA, Diagnostic accuracy, Chronic kidney disease, Biomarker

## Abstract

**Background:**

Accurate detection of kidney damage is key to preventing renal failure, and identifying biomarkers is essential for this purpose. We aimed to assess the accuracy of miRNAs as diagnostic tools for chronic kidney disease (CKD).

**Methods:**

We thoroughly searched five databases (MEDLINE, Web of Science, Embase, Scopus, and CENTRAL) and performed a meta-analysis using R software. We assessed the overall diagnostic potential using the pooled area under the curve (pAUC), sensitivity (SEN), and specificity (SPE) values and the risk of bias by using the QUADAS-2 tool. The study protocol was registered on PROSPERO (CRD42021282785).

**Results:**

We analyzed data from 8351 CKD patients, 2989 healthy individuals, and 4331 people with chronic diseases. Among the single miRNAs, the pooled SEN was 0.82, and the SPE was 0.81 for diabetic nephropathy (DN) vs. diabetes mellitus (DM). The SEN and SPE were 0.91 and 0.89 for DN and healthy controls, respectively. miR-192 was the most frequently reported miRNA in DN patients, with a pAUC of 0.91 and SEN and SPE of 0.89 and 0.89, respectively, compared to those in healthy controls. The panel of miRNAs outperformed the single miRNAs (pAUC of 0.86 vs. 0.79, *p* < 0.05). The SEN and SPE of the panel miRNAs were 0.89 and 0.73, respectively, for DN vs. DM. In the lupus nephritis (LN) vs. systemic lupus erythematosus (SLE) cohorts, the SEN and SPE were 0.84 and 0.81, respectively. Urinary miRNAs tended to be more effective than blood miRNAs (*p* = 0.06).

**Conclusion:**

MiRNAs show promise as effective diagnostic markers for CKD. The detection of miRNAs in urine and the use of a panel of miRNAs allows more accurate diagnosis.

**Supplementary Information:**

The online version contains supplementary material available at 10.1186/s12882-024-03702-y.

## Key Learning Points

1. The diagnostic performance of miRNAs in chronic kidney diseases needs to be investigated, as previous systematic reviews and meta-analyses are scarce, and individual cohort studies have confirmed their importance in kidney pathophysiology.

2. Using multiple miRNAs as biomarkers can lead to more precise CKD diagnosis, as they outperform single miRNAs compared to healthy and people with chronic disease groups. The overall diagnostic accuracy of urinary miRNAs tended to be greater than that of blood samples, suggesting that they are noninvasive and readily available diagnostic markers in CKD patients.

3. Incorporating miRNAs as diagnostic markers for CKD in combination with traditional markers might improve the accuracy and efficiency of diagnosis. To optimize miRNA diagnostic performance, it is essential to consider the various biological sample types, comparison groups (control), and different kidney diseases.

**Supplementary Information:**

The online version contains supplementary material available at 10.1186/s12882-024-03702-y.

## Introduction

By 2040, chronic kidney disease (CKD) is expected to be the fifth most common cause of death worldwide [1], raising awareness of the need for sensitive diagnostic biomarkers and novel therapeutic strategies. To assess the degree of renal damage, monitor disease progression, and evaluate the response to treatment, kidney function should be assessed by the estimated glomerular filtration rate (eGFR) from the serum creatinine level or by measuring the degree of proteinuria as a standard diagnostic procedure. However, controversy still exists regarding the staging, diagnosis, and prediction of complications in CKD [2].

MicroRNAs (miRNAs), the largest family of short noncoding RNAs, have recently emerged as potential diagnostic markers and therapeutic agents [3, 4]. Initial studies were conducted in 2004 and 2005 to identify miRNAs specific to or enriched in the kidney [5, 6]. The significance of miRNAs in kidney function was revealed in 2007 [7], leading to the implementation of the Human MicroRNA Disease Database [8] and a subsequent increase in research evidence. However, the diagnostic performance of miRNAs in CKD needs to be investigated, as preclinical studies have confirmed their importance in kidney pathophysiology [9]. In addition, miRNAs exhibit superior stability in degraded RNA samples, which makes them more suitable biomarkers [10].

Furthermore, miRNAs may outperform conventional markers; for instance, a pilot study demonstrated that sets of miRNAs show alterations days before an increase in the serum creatinine concentration [11]. Serum and urine miRNAs are used for diagnostic purposes in patients with CKD [11–13], and some miRNAs may be specific to the disease or to the sample during kidney damage. The sensitivity (SEN), specificity (SPE), positive predictive value (PPV), and negative predictive value (NPV) are the most common values used to estimate diagnostic accuracy. However, for more accurate analysis, it is essential to consider the receiver operating characteristic (ROC) curve and the area under the curve (AUC) for overall diagnostic accuracy.

We aimed to assess the accuracy of single and panel miRNAs for diagnosing kidney disease using various sample types by using receiver operating characteristic (ROC) analysis and area under the curve (AUC) analysis. We also investigated the diagnostic performance of miRNAs by comparing patients with overt kidney disease groups with healthy individuals and chronic disease groups (such as DM patients and SLE patients without nephropathy or patients with different kidney diseases).

## Methods

The study protocol was registered in the PROSPERO International Prospective Register of Systematic Reviews (CRD42021282785). No ethical approval was needed, as all the data were previously published in peer-reviewed journals, and no patients were involved in the study’s design, conduct, or interpretation. We report our study based on the recommendation of the Preferred Reporting Items for Systematic Reviews and Meta-Analyses (PRISMA) 2020 guidelines [14] (Supplementary Table S1), while we followed the Cochrane Handbook (version 6.2) [15].

### Eligibility criteria

To define our clinical question and eligibility criteria, we applied the PIRD framework as follows: the population (P) consisted of individuals with (case group) and without CKD (healthy and chronic disease groups); the index test (I) was miRNA detection performed by qRT‒PCR; the reference test (R) was clinical diagnosis confirmed by biopsy or laboratory parameters; and the diagnosis (D) was chronic kidney disease. Studies were considered eligible if they met the following criteria: (1). The diagnostic accuracy of miRNA for CKD was provided; (2) all patients with CKD were diagnosed by the gold standards in diagnostics (biopsy or laboratory); (3) AUC, ROC curve, SEN and SPE were provided; (4) observational and interventional studies were included.

Studies were excluded from the systematic meta-analysis review if (1) the inclusion criteria were not met, (2) data on outcomes of interest were impossible to extract from the published results and upon request from the corresponding author, (3) the exposed group included acute kidney injury, CKD with coronary artery calcification, congenital kidney diseases, polycystic kidney disease, or Alport syndrome, (4) the study design included reviews, meta-analyses, case series and reports or letters, or (5) overlapping studies.

### Search strategy and selection process

The Cochrane Central Register of Controlled Trials (CENTRAL), Web of Science, Embase, Scopus, and MEDLINE (via NCBI PubMed) databases were screened using an electronic search strategy (see Supplementary Methods). The first search was conducted up to November 25, 2021, and updated by the second search on November 26, 2022. There were no language restrictions or filters imposed. The references used were managed in EndNote 20 (Clarivate Analytics, Philadelphia, PA, USA). After removing duplicates automatically and manually, two independent investigators performed article selection based on predefined eligibility criteria in a two-step manner—first considering the title and abstract and, subsequently, the full-text content. Cohen’s kappa index was calculated at each selection step. A third investigator resolved disagreements. The reference lists of the included studies were also assessed for additional eligible reports.

### Data collection

Two independent investigators performed the data collection. Disagreements were resolved by consensus. The following data were extracted: study characteristics (first author, publication year, country, study population), eligibility criteria, definition of case and control groups, definition of kidney disease, stages of CKD (as defined in each study either by the histology score or laboratory parameters or eGFR), sample number and type. The following variables were extracted for the purposes of miRNA diagnostic accuracy: miRNA name, miRNA detection, normalization method, fold change, AUC value, cutoff point for the ROC curve, overall diagnostic accuracy, SEN, SPE, and correlation coefficient between miRNA expression and laboratory parameters, if available. Standardized Microsoft Excel sheets (Microsoft Office 365, Redmond, WA, USA) were used for data collection. In the case of missing data, the study authors were contacted for retrieval. WebPlotDigitizer [16] was used to extract the area under the curve (AUC), negative value (SE), and specificity (SPE) values if only the ROC curves were available instead of the abovementioned statistics.

### Data synthesis and analysis

In terms of clinical applicability, the data were pooled, taking into account the following moderators: (1) comparison of CKD patients with healthy individuals and people with chronic diseases, (2) individual kidney diseases, (3) biological sample types, (4) single miRNAs or panel miRNAs, and (5) ethnicity. We analyzed the most frequent miRNAs in the included studies separately. The statistical analysis was performed using R software (version 4.1.2.) [17]. A p value less than 0.05 indicated statistical significance. We collected AUC values and computed their standard deviation using the confidence interval or the method of Hanley and colleagues [18] when the confidence interval was not available. For rigorous pAUC values, we considered the correlations between sample errors and random effects corresponding to the miRNAs present in the same study by a multivariate mixed-effect model supplemented with the robust approach [19]. Univariate and multivariate analyses of AUC values were performed to determine the effect of moderator variables. In addition, we applied well-known methods [20–22] to obtain pooled SEN and SPE. To address these correlations, we randomly selected SEN and SPE; we chose only one miRNA from each study and then calculated the pooled SEN and SPE. According to the ROC plot visualization, the size of the prediction region provided insight into heterogeneity.

In the case of the meta-analysis of specific miRNAs, we performed classical inverse variance AUC meta-analysis due to the lack of the abovementioned correlations. In these cases, the heterogeneity was calculated by I^2^. We created AUC funnel plots showing all available data to assess publication bias. Similarly, as above, we performed Egger’s test after randomly selecting one result from each study. Studies reporting only CKD without a specific diagnosis were excluded from the meta-analysis to avoid selection bias. For a detailed description of the statistical analysis, see the Supplementary Methods section.

### Risk of bias and quality assessment

Three independent investigators (D.G., U.N.D.T., and G.G.) assessed the risk of bias in each study. The details are provided in the Supplementary Material. The Quality Assessment of Diagnostic Accuracy Studies (QUADAS-2) [23] guidelines were used to evaluate study quality and assess the following variables: patient selection, index test, reference standard, and flow and timing (see Supplementary Material). Each variable was evaluated for risk of bias, and the first three domains were evaluated for applicability. The answer to each question was “yes,” “no,” or “unclear.” A “yes” response indicated a low likelihood of bias, whereas a “no” or “unclear” response indicated the opposite.

## Results

### Search and selection

Eighty-seven studies met the eligibility criteria, and all were included in the meta-analysis [24–106]. All included records were reported as peer-reviewed full-text publications. The study selection process is described in detail in Supplementary Fig. 1.

**Fig. 1:**
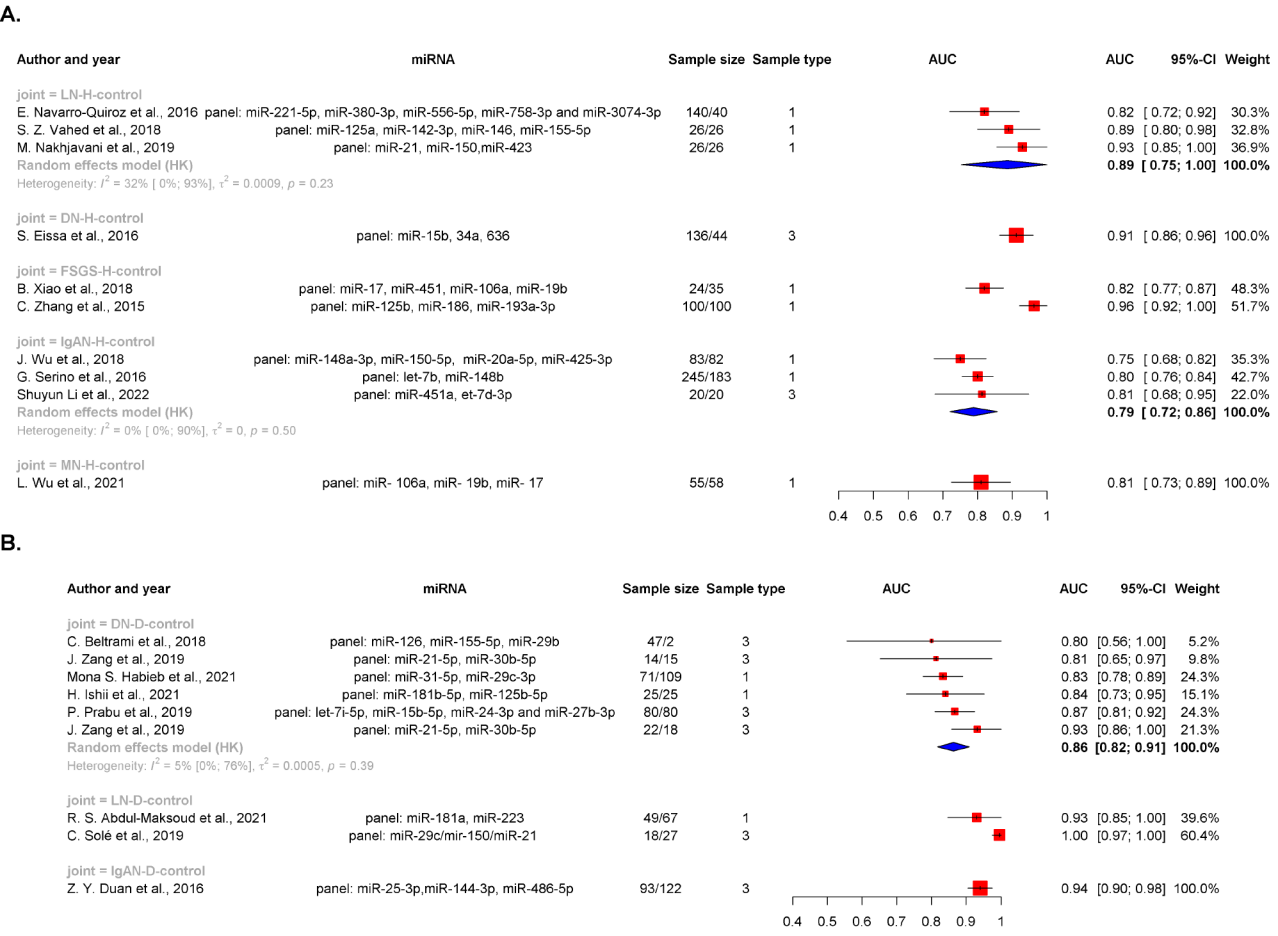
The pooled AUC values of panel miRNAs in CKD patients compared with (**A**) healthy controls and (**B**) people with chronic diseases. Legend: Pooled AUC values (≥ 3 study) for panel miRNAs in patients with different kidney diseases; (**A**) healthy and (**B**) chronic disease groups stratified by sample type. Sample sizes are indicated as case/control groups. Sample types are illustrated by 1 (blood) and 3 (urine) abbreviations: AUC, area under the curve; 95% CI, 95% confidence interval; H, healthy individuals; D, people with chronic diseases; DN, diabetic nephropathy

### Study characteristics

Eighty-seven articles (from 2013 to 2022) reported 238 single and 34 panel miRNA results, including 8351 patients with kidney diseases, 2989 healthy individuals and 4331 patients with SLE and diabetes mellitus (DM), e.g., as a control population (see Supplementary Table S2). Forty-seven studies were conducted with patients with CKD vs. healthy controls, and 40 were conducted with patients with CKD vs. people with chronic diseases (patients with SLE or T2DM without kidney disease or patients with different kidney diseases; see Supplementary Table S2). Several studies reported more than one kidney disease. All the studies used quantitative real-time reverse transcription PCR (qRT‒PCR) to detect miRNAs. Regarding biological sample types, 28 studies detected miRNAs in urine, 56 in blood (15 in blood, 4 in PBMCs, 18 in plasma, 20 in serum, and two in serum exosomes), and 3 in kidney samples. Eighty-seven studies reported the pAUC values for single and panel miRNAs (Supplementary Table S3). The summary ROC curve was reported in 77 studies for single miRNAs and 20 studies for panel miRNAs. In total, 71 studies were included in the meta-analysis. The characteristics of the included studies are presented in Supplementary Table S4.

### Area under the curve values

The healthy control studies had an overall pAUC of 0.84 (95% CI = 0.81–0.87), while the chronic disease studies had a pAUC of 0.80 (95% CI = 0.77–0.82). The difference between the two control groups was significant (*p* = 0.01) (Supplementary Figure S2). We performed all the other analyses separately for the healthy and chronic disease groups.

The results from univariate and multivariate analyses agreed, except for the findings related to the sample type moderator. In the healthy and chronic disease groups, only the effect of the panel miRNA variable was significant, with pAUC values of 0.88 and 0.91, respectively (Supplementary Figure S3).

Moreover, there was no significant difference between urine and blood sample types in studies that included people with chronic diseases. However, according to our multivariate analysis, urine tended to have a slightly greater AUC than blood (*p* = 0.06) (Table 1). Unfortunately, kidney biopsy sample data were unavailable for multivariate analysis, but the overall pAUC was 0.82 for kidney diseases compared to people with chronic diseases (Supplementary Figure S4).

**Table 1:** Multivariate analysis of AUC values (panel miRNA – sample - disease interaction model with healthy control groups)

Coefficient	Estimate	SE	t-stat	d.f. (Satt)	*p*-val (Satt)	Sig.
Reference	0.84	0.02	34.95	16.67	< 0.001	***
Sample: Urine	0.05	0.03	2.05	18.05	0.06	.
Disease: Focal segmental glomerulosclerosis	-0.07	0.05	-1.41	6.27	0.21	
Disease: Lupus nephritis	-0.03	0.03	-0.83	19.92	0.42	
Disease: membranous nephropathy	-0.06	0.06	-1.12	4.39	0.32	
Disease: IgA nephropathy	-0.08	0.05	-1.85	9.3	0.10	.
Panel miRNA	0.08	0.02	4.61	8.88	0.001	**

Legend: contains the multivariate analysis of the panel – sample type - disease interaction in the model with healthy control groups. The reference variables were diabetic nephropathy (DN) – sample type: blood - single miRNAs. Significance: *p* < 0.001***, *p* < 0.01**, *p* > 0.05*.

Among the DN patients and healthy controls, only the area under the curve (AUC) values for a panel of miR-15b, miR-34a, and miR-636 were reported (0.91, 95% CI = 0.86–0.96) (Fig. 1), and the pAUC for a single miRNA was 0.86 (95% CI = 0.82–0.89) (Supplementary Figure S5).

The pAUC of DN vs. DM was 0.86 (95% CI = 0.82–0.91) for the panel miRNAs (Fig. 1) and 0.80 (95% CI = 0.76–0.83) for the single miRNAs (Supplementary Figure S5). Among the individual studies, the miR-21 and miR-30-b-5p panels had higher AUCs in DN patients than in DM patients (0.93; 95% CI = 0.86-1.0) (Fig. 1).

The LN patients in the SLE control group had a greater AUC than did those in the other kidney disease group, e.g., the panel containing miR-29c, miR-150, and miR-21 had an AUC of 1.00 (95% CI = 0.97-1.00) (Fig. 1).

The pAUC of the panel of miRNAs in IgAN patients vs. healthy controls was 0.79 (95% CI = 0.72–0.86) (Fig. 1).

Overall, the moderator variables were highly interdependent and affected the interpretation of the results. To eliminate some of the dependencies between the moderators, we omitted ethnicity from the multivariate analysis (Tables 1 and 2) but conducted univariate analyses (Supplementary Figure S6).

**Table 2:** Multivariate analysis of AUC values (panel miRNA – sample - disease interaction model with diseased control groups)

Coefficient	Estimate	SE	t-stat	d.f (Satt)	*p*-val (Satt)	Sig.
Reference	0.78	0.02	40.45	23.27	< 0.001	***
Sample: Urine	0.01	0.03	0.32	22.54	0.75	
Disease: Lupus nephritis	0.03	0.03	1.23	8.42	0.25	
Disease: IgA nephropathy	-0.004	0.08	-0.05	2.43	0.97	
Panel miRNA	0.08	0.01	6.13	6.46	< 0.001	***

Legend: Table 1 contains the multivariate analysis of when panel – sample type - disease interaction in the model with diseased control groups. The reference variables are Diabetic nephropathy (DN) – sample type: blood - single miRNAs. Significance; *p* < 0.001 ***, *p* < 0.01**, *p* > 0.05*

**Fig. 2:**
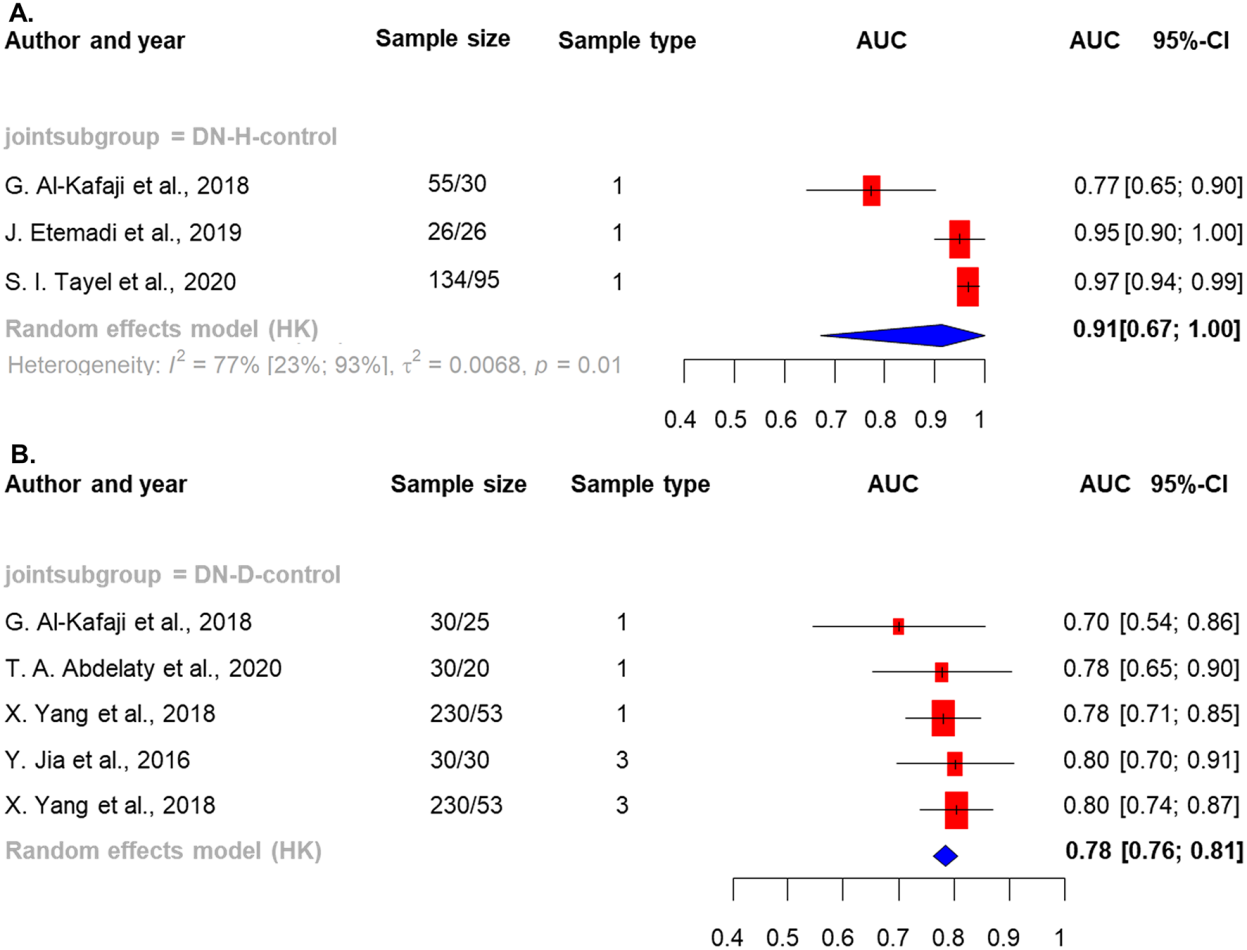
The pooled AUC values of miR-192 in the diabetic nephropathy group compared with those in the (**A**) healthy controls and (**B**) chronic disease groups (diabetes mellitus). Legend: The pooled AUC values of miR-192 in diabetic nephropathy (DN) patients compared to those in (**A**) healthy individuals and (**B**) diabetes mellitus patients. Sample sizes are indicated as case/control groups. Sample types are illustrated by 1 (blood) and 3 (urine) abbreviations: AUC, area under the curve; 95% CI, 95% confidence interval; H, healthy indivdiual; D, chronic disease group; DN, diabetic nephropathy

The pAUC values of the most frequently reported single miRNAs, miR-192, miR-21, and miR-146a, were analyzed. In contrast, compared with those of healthy individuals and T2DM patients without nephropathy, the pAUC of miR-192 was 0.91 (95% CI = 0.67-1.0), and the pAUC was 0.78 (95% CI = 0.76–0.81) for the diagnosis of DN (Fig. 2). miR-146a had a greater pAUC (0.92; 95% CI = 0.70-1.0) in LN patients than in healthy controls (0.60; 95% CI = 0.43–0.77) in LN patients vs. SLE patients (Supplementary Figure S7).

### Sensitivity and specificity in studies with healthy controls

**Fig. 3:**
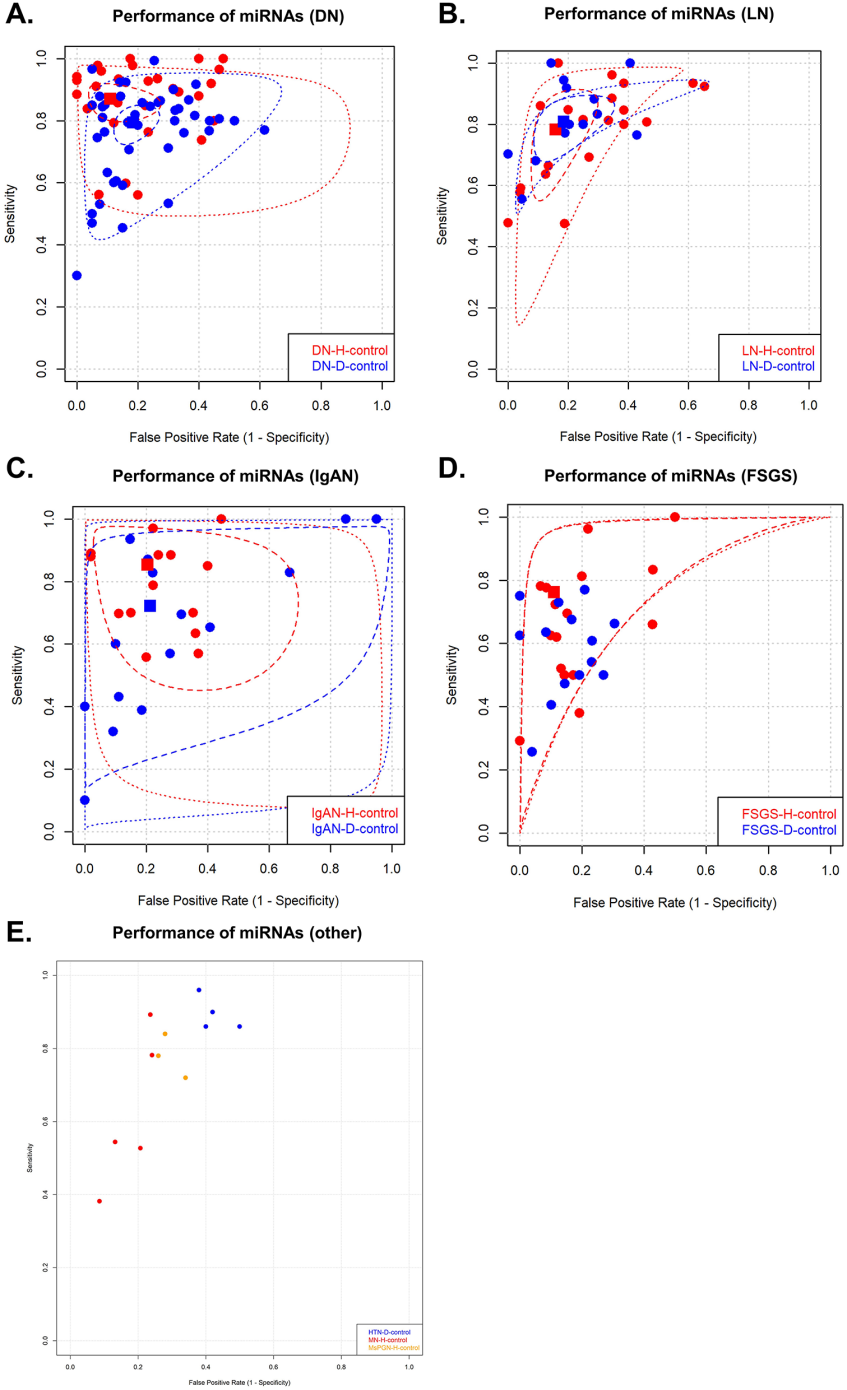
The pooled sensitivity and specificity of single miRNAs in CKDs. Legend: Representative ROC curves of the sensitivity and specificity of single miRNAs are shown for kidney diseases; (**A**) Diabetic nephropathy (DN), (**B**) Focal segmental glomerulosclerosis (FSGS), (**C**) IgA nephropathy (IgAN), (**D**) Lupus nephritis (LN) and (**E**) other kidney diseases. The pooled sensitivity and specificity are pooled if there are more than four studies and visualized by squares: red – healthy individuals and blue–chronic disease groups. Other kidney diseases include hypertensive nephropathy (HTN), membranous nephropathy (MN), and mesangial proliferative glomerulonephritis (MsPGN)

**Fig. 4:**
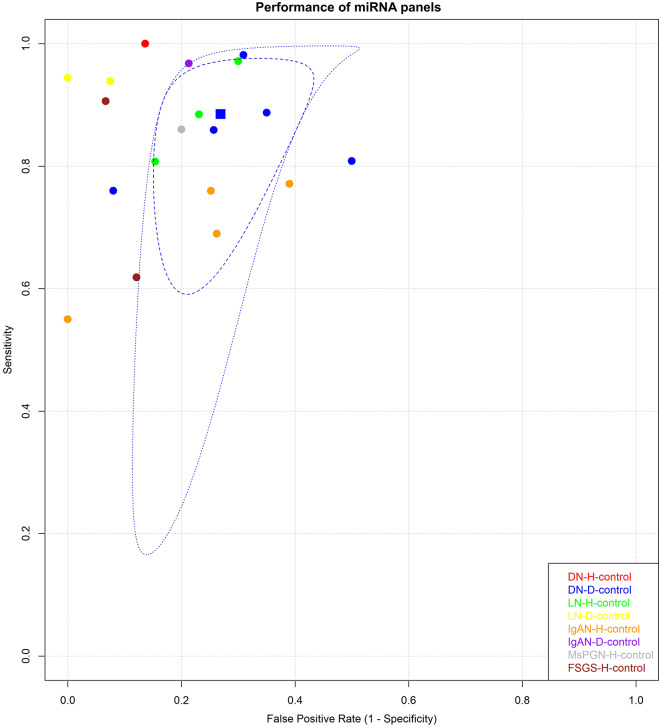
The pooled sensitivity and specificity of the panel of miRNAs for CKDs. Legend: ROC curves of sensitivity and specificity for panel miRNAs are shown for kidney disease. The pooled sensitivity and specificity (≥ 4 studies) are shown for diabetic nephropathy compared to type 2 diabetes mellitus (DN-Pool2). Abbreviations: DN-Pool1, diabetic nephropathy compared to healthy controls; LN-Pool1, lupus nephritis compared to healthy controls; LN-Pool2, lcontrol, IgAN compared to systematic lupus erythematosus; IgAN-Pool2; IgA nephropathy compared to healthy controls; IgAN-Pool1, IgA nephropathy compared to chronic disease groups; NS-Pool1, nephrotic syndrome compared to healthy controls; MsPGN-Pool1, membranoproliferative glomerulopathy compared to healthy controls; FSGS-Pool2, focal segmental glomerulosclerosis compared to chronic disease group

For single miRNAs, the pooled SEN and SPE for DNs compared to those for healthy controls were 0.91 (95% CI = 0.86s-0.95) and 0.89 (95% CI = 0.77–0.95), respectively (Fig. 3A). In patients with LN, the pooled SEN was 0.81 (95% CI = 0.68–0.90), and the SPE was 0.80 (95% CI = 0.72–0.87) (Fig. 3B). The pooled analysis of other kidney diseases is shown in Fig. 3C-E. Among the included studies, miR-192 was most frequently reported in DN patients compared to healthy controls, with a pooled SEN of 0.89 (95% CI = 0.82–0.94) and SPE of 0.89 (95% CI = 0.72–0.96) (Fig. 4).

### Sensitivity and specificity in studies including people with chronic diseases

**Fig. 5:**
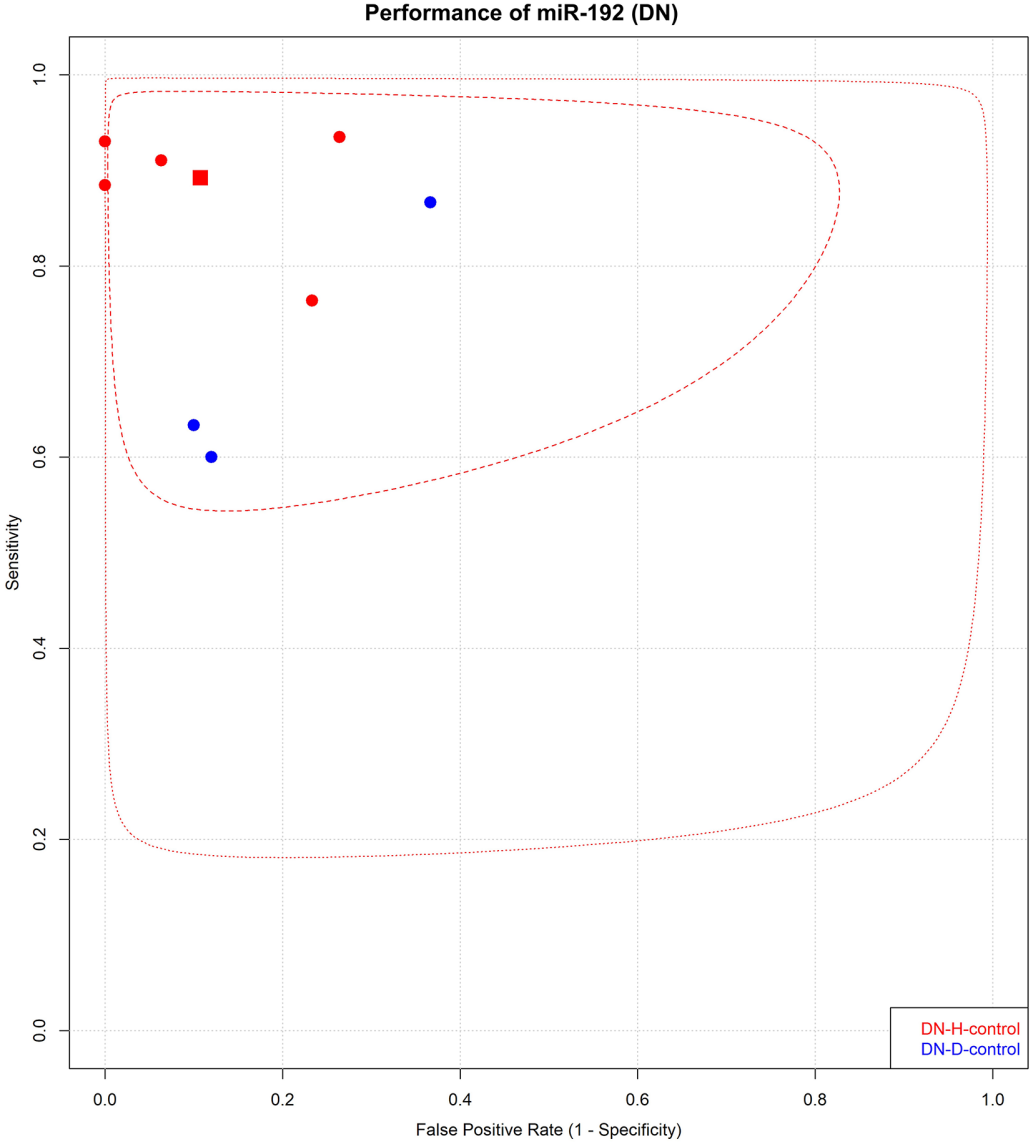
The pooled sensitivity and specificity of miR-192 in diabetic nephropathy. Legend: The pooled sensitivity and specificity of miR-192 in diabetic nephropathy (DN) patients are shown. A square illustrates the summary pooled point available. Circles indicate individual sensitivity and specificity. Abbreviations: DN-Pool1, diabetic nephropathy compared to healthy control; DN-Pool2, diabetic nephropathy compared to type 2 diabetes

The pooled SEN and SPE values for single miRNAs in DN patients compared to those in T2DM patients were 0.82 (95% CI = 0.76–0.87) and 0.81 (95% CI = 0.74–0.86), respectively (Fig. 3A). When the LN patients were compared to the SLE patients, the pooled SEN was 0.84 (95% CI = 0.74–0.91), and the SPE was 0.81 (95% CI = 0.72–0.88) (Fig. 3B). Figure 3C-E shows the pooled analysis of other kidney diseases. Among the panel miRNAs, the pooled SEN and SPE were 0.89 (95% CI = 0.78–0.94) and 0.73 (95% CI = 0.66–0.79), respectively, for DN compared with patients with T2DM (Fig. 5).

### Sensitivity analysis for meta-analysis

The different analyses served as sensitivity analyses for each other. Moreover, we repeated the procedures with different correlation imputations to account for correlations. In the case of random selection, we repeated the process several times with roughly the same results.

### Publication bias

If there were more than ten articles, a publication bias analysis was conducted and made available for DN, LN, IgAN, FSGS, and MN studies (Supplementary Figures S8-16). As a result, only LN studies with SLE control groups had no publication bias (Egger’s test, *p* = 0.09) (Supplementary Figure S10).

### Risk of bias assessment

Supplementary Figure S17 and Table S5 of the QUADAS-2 assessment show that all CKD patients included in the studies were diagnosed according to clinically proven diagnostic criteria. All the studies had case‒control or cohort designs, which may have introduced high or unclear risks to the selection field.

## Discussion

To our knowledge, this is the first comprehensive systematic review and meta-analysis investigating the diagnostic accuracy of miRNAs in different biological samples and control groups for several types of kidney diseases. In our study, we found that the panel of miRNAs more effectively detected kidney diseases than did the single miRNAs. Furthermore, regarding the overall diagnostic performance of the miRNAs, the pAUC was better when comparing kidney disease patients to healthy controls than when comparing chronic disease groups. In addition, urinary miRNAs generally showed greater diagnostic accuracy than blood samples, with an overall pAUC of 0.86 and an overall pAUC of 0.82, respectively.

Previous studies have confirmed that the diagnostic accuracy of a panel of miRNAs is superior to that of single miRNAs [107]. We also considered specific kidney diseases and conducted separate analyses for the healthy and chronic disease groups while considering the correlation between results from the same population. In addition, we performed a pooled analysis of the area under the curve (AUC) from each study to determine the overall diagnostic accuracy, eliminating the possibility of threshold effects.

We found that certain panels of miRNAs had higher overall AUCs when comparing DN patients, LN patients, or FSGS patients to healthy controls. These panels included miR-15b, miR-34a, and miR-636 for DN; miR-21, miR-150, and miR-423 for LN; and miR-125b, miR-186, and miR-193a-3p for FSGS. Additionally, in disease control studies, panels of miRNAs, such as miR-21 and miR-30-b-5p, had better overall AUCs for differentiating between DN patients and T2DM patients. Similarly, panels of miR-29c, miR-150, and miR-21 effectively distinguished LN from SLE patients. Another study by J. Li and colleagues identified specific miRNA panels that were superior for CKD, such as miR-27b-3p and miR-1228-3p for DN; miR-21, miR-150, and miR-29c for LN; and miR-106a-5p and miR-30a-5p for mesangial proliferative glomerulonephritis (MsPGN) [107].

In comparison, our findings may differ slightly from those of J. Li and colleagues [107]; therefore, it is essential to note that our analysis included separate controls for healthy and diseased individuals with different kidney diseases (details in Supplementary Table S2). Multiple studies on cancer have shown that a miRNA panel is a more effective diagnostic marker than a single miRNA [108]. For instance, V. Bhaskaran and colleagues reported that several miRNAs exhibit a clustered expression pattern in glioblastoma, even if they are not encoded within the same genetic locus [109]. Moreover, these studies focused on targeting several miRNAs simultaneously; as a result, coexpressed miRNAs repress epigenetic oncogenic signaling pathways and have more profound therapeutic effects than single-miRNA therapy [109]. In nephrology, it might also be helpful to consider the expression of miRNA clusters and test a miRNA panel for diagnostic purposes.

In our included studies, several miRNAs exhibited high sensitivity and specificity (over 90%). These included miR-451 for the diagnosis of DN vs. DM [25] and miR-126 [106], miR-21 [43], miR-636 [38], and miR-192 [81, 92] for the diagnosis of DN vs. healthy controls. miR-142a-5p effectively differentiated nephrotic syndrome (NS) patients from healthy individuals [32]. In addition, J. Li and colleagues discovered that miR-133, miR-30a, and miR-126 were promising markers for the diagnosis of CKD with high accuracy levels of sensitivity or specificity above 90% [107].

These miRNAs are validated in preclinical studies, and their molecular function is reviewed by Mahtal and colleagues [110]. For example, miR-192 mediates TGF-beta/Smad3-driven renal fibrosis in a mouse model of UUO and a rat remnant kidney model [111]. miR-451 might decrease inflammation in DN by inhibiting the synthesis of the NF-κB effector proteasome subunit-β type-8 [112]. Silencing miR-21 protects animal models from DKD [113] as its overexpression is associated with mesangial cell hypertrophy by regulating Cdc25a and Cdk6, podocyte damage, and ECM accumulation by targeting Pten [113]. miR-126 supports kidney recovery after acute injury by promoting vascular integrity and association with stromal cell-derived factor 1/CXCR4-dependent vasculogenic progenitor cell mobilization [114].

While the results compared to those of healthy controls looked promising, we recommend comparing kidney disease patients with chronic disease groups. This approach could provide significant clinical benefits in distinguishing, for instance, between patients with DN and those with T2DM without nephropathy. Research on miRNAs is still in its early stages, which can lead to inconsistencies in the expression of specific miRNAs measured in laboratories within the same sample type and disease [115]. Therefore, comparing chronic disease and healthy control groups in different biological samples helps to identify potential diagnostic miRNA biomarkers.

In line with only one existing meta-analysis [107], urinary miRNAs tended to perform better than blood miRNAs in our meta-analysis, although the difference did not reach statistical significance (multivariate *p* = 0.06). The quantity of miRNAs in cell-free urine is less than in plasma or urinary exosomes because of the high RNase activity present in the kidney, bladder, and urinary tract [116]. Urine miRNAs could originate from cells in the urinary tract or could be filtered from plasma, indicating kidney-related or systemic diseases. Given these points, plasma miRNAs may be more reliable for diagnosing CKD, and comprehensive cohort studies using various biological samples can yield more definitive findings. Nevertheless, urine tests might be the most applicable test in almost all clinical settings. Several urinary miRNAs had higher AUCs (0.98–0.99); in the included studies (miR-204, miR-636, and miR-146a), these miRNAs might be potential diagnostic biomarkers in CKD. Urinary miRNAs generally perform better in CKDs because they correlate best with kidney tissue [117] and pathological consequences and are surprisingly stable [118]. Urine samples had a greater overall AUC for all kidney diseases except DN than did healthy controls. For DN patients, blood samples performed better than urine samples did, with a pAUC of 0.87 compared to 0.84. However, further investigations are needed to understand the underlying mechanisms involved.

To our knowledge, only a few studies compare the diagnostic accuracy of miRNAs with conventional markers in prospective cohort studies for CKD, but none for the overall disease course, starting from the early to late stages. They mostly reported an association between miRNA expression and conventional markers of CKD in addition to survival prediction, cardiovascular outcomes, and kidney disease progression [94, 119–121]. For example, the large cohort of 601 CKD patients showed that reduction of miR-126 and miR-223 are associated with a lower eGFR but cannot independently predict survival, cardiovascular, and kidney function decline [119].

However, combining miRNAs with clinical parameters has been suggested to be a more effective diagnostic biomarker than miRNAs alone [122]. For example, Miller and colleagues reported that a model combining urinary particle concentrations, blood lactate levels, urinary extracellular vesicles positive for PODXL, and miR-125a-5p could predict kidney injury [13].

Due to the limited number of available studies, it was not feasible to perform a meta-analysis on NS, MsPGN, or hypertensive nephropathy (HTN); only systematic reviews and visualizations were included. However, we suggest that future studies investigate the diagnostic accuracy of NS-, MsPGN-, and HTN-specific miRNAs. For instance, studies in a systematic review showed that miR-142a-5p has a better diagnostic performance than the panel miRNAs miR-30a-5p and miR-181a-5p in childhood idiopathic nephrotic syndrome [32].

Unfortunately, most of those studies did not report disease stages, so we could not analyze disease stage. Diagnostic studies must distinguish between early and late CKD stages for accurate analysis of biomarkers, particularly miRNAs. Although further research is needed to develop the most effective approach, preliminary findings show that using a miRNA panel in CKD patients can significantly enhance diagnostic accuracy.

### Strengths and limitations of the study

Our analysis has several advantages that are worth highlighting. First, we attempted to determine the diagnostic accuracy of miRNAs in different types of chronic kidney diseases with various sample types. Second, by performing a subgroup analysis, we examined the potential of miRNAs in differentiating between healthy and chronic disease groups. From a statistical perspective, we can list the following advantages of our meta-analysis of diagnostic accuracy. Given the complexity of miRNAs, the different threshold values used for background and fold change expression, we considered the area under the curve (AUC) from each study for overall diagnostic accuracy and obtained pAUC values. The threshold value does not influence these values and provides more reliable results than pooled SEN and SPE results. To avoid overestimating pooled results, we considered correlations between variables in studies that reported multiple miRNAs in the same population.

Unfortunately, we were unable to assess differences in the accuracy of miRNA detection at different CKD stages due to a lack of data. Importantly, many studies in this field are retrospective case‒control studies that can potentially introduce bias in assessing the quality of patient selection domains. The miRNA detection and normalization methods varied also in the included studies in our meta-analysis. There is no universal agreement on the cutoff values of reference genes for miRNA studies, which may lead to inconsistent results in the relative quantitative analysis of miRNAs (Table S6). This limitation should be taken into account by researchers when interpreting meta-analysis results. Furthermore, it is essential to conduct large prospective cohort studies to address these limitations and expedite the integration of miRNAs into clinical applications.

### Implications for practice and research

Based on our results, we propose the use of a panel of miRNAs to distinguish DN patients from T2DM patients without nephropathy and LN from SLE patients without kidney disease. To incorporate our findings into everyday medical practice [123], it is necessary to conduct prospective, well-designed cohort studies. These studies should assess the accuracy of both single and panel miRNAs for diagnosing CKDs at various stages and compare them to traditional biomarkers.

## Conclusion

Analysis of miRNAs can distinguish between CKD patients, healthy individuals, and diseased patients without overt CKD, such as T2DM patients and SLE patients. Using miRNA panels for diagnosis is more effective than relying on a single miRNA. Additional cohorts should be evaluated for the diagnostic performance of miRNAs in the early and late stages of CKDs.

### Electronic supplementary material

Below is the link to the electronic supplementary material.

Supplementary Material 1

Supplementary Material 2

## Data Availability

Data is provided within the manuscript and supplementary files.

## Abbreviations

AUC: Area under the curve
CKD: Chronic kidney disease
DN: Diabetic nephropathy
Fig: Figure: Fig. S: Supplementary Figure
HTN: Hypertensive nephropathy
IgAN: IgA nephropathy
FN: False negative
FP: False positive
FSGS: Focal segmental glomerulosclerosis
eGFR: Estimated glomerular filtration rate
MN: Membranous nephropathy
miRNAs: MicroRNAs
MsPGN: Mesangial proliferative glomerulonephritis
pAUC: Pooled area under the curve
SEN: Sensitivity, specificity
SLE: Systematic lupus erythematosus
SPE: Specificity
T2DM: Type 2 diabetes mellitus
PBMC: Peripheral blood mononuclear cell
PODXL: Podocalyxin
PRISMA: Preferred Reporting Items for Systematic Reviews and Meta-Analyses
ROC: Receiver operating characteristic
SROC: Summary receiver operating characteristic

## References

[1] Foreman KJ, et al. Forecasting life expectancy, years of life lost, and all-cause and cause-specific mortality for 250 causes of death: reference and alternative scenarios for 2016-40 for 195 countries and territories. Lancet. 2018;392(10159):2052–90. 30340847 10.1016/S0140-6736(18)31694-5 PMC6227505

[2] Polkinghorne KR. Controversies in chronic kidney disease staging. Clin Biochem Rev. 2011;32(2):55–9. 21611077 PMC3100281

[3] Trionfini P, Benigni A, Remuzzi G. MicroRNAs in kidney physiology and disease. Nat Rev Nephrol. 2015;11(1):23–33. 25385286 10.1038/nrneph.2014.202

[4] Sridharan K, Gogtay NJ. Therapeutic nucleic acids: current clinical status. Br J Clin Pharmacol. 2016;82(3):659–72. 27111518 10.1111/bcp.12987 PMC5338117

[5] Sun Y, et al. Development of a micro-array to detect human and mouse microRNAs and characterization of expression in human organs. Nucleic Acids Res. 2004;32(22):e188. 15616155 10.1093/nar/gnh186 PMC545483

[6] Naraba H, Iwai N. Assessment of the microRNA system in salt-sensitive hypertension. Hypertens Res. 2005;28(10):819–26. 16471176 10.1291/hypres.28.819

[7] Kato M, et al. MicroRNA-192 in diabetic kidney glomeruli and its function in TGF-beta-induced collagen expression via inhibition of E-box repressors. Proc Natl Acad Sci U S A. 2007;104(9):3432–7. 17360662 10.1073/pnas.0611192104 PMC1805579

[8] Huang Z, et al. HMDD v3.0: a database for experimentally supported human microRNA-disease associations. Nucleic Acids Res. 2019;47(D1):D1013–7. 30364956 10.1093/nar/gky1010 PMC6323994

[9] Lee EC, et al. Discovery and preclinical evaluation of anti-mir-17 oligonucleotide RGLS4326 for the treatment of polycystic kidney disease. Nat Commun. 2019;10(1):4148. 31515477 10.1038/s41467-019-11918-y PMC6742637

[10] Hall JS, et al. Enhanced stability of microRNA expression facilitates classification of FFPE tumour samples exhibiting near total mRNA degradation. Br J Cancer. 2012;107(4):684–94. 22805332 10.1038/bjc.2012.294 PMC3419950

[11] Aguado-Fraile E, et al. A pilot study identifying a set of microRNAs as precise diagnostic biomarkers of Acute kidney Injury. PLoS ONE. 2015;10(6):e0127175. 26079930 10.1371/journal.pone.0127175 PMC4469584

[12] Wang J, et al. Serum miR-21 may be a potential Diagnostic Biomarker for Diabetic Nephropathy. Exp Clin Endocrinol Diabetes. 2016;124(7):417–23. 26575121 10.1055/s-0035-1565095

[13] Miller D, et al. Urinary extracellular vesicles and micro-RNA as markers of acute kidney injury after cardiac surgery. Sci Rep. 2022;12(1):10402. 35729178 10.1038/s41598-022-13849-z PMC9213448

[14] Page MJ, et al. The PRISMA 2020 statement: an updated guideline for reporting systematic reviews. BMJ. 2021;372:n71. 33782057 10.1136/bmj.n71 PMC8005924

[15] Higgins JPT, Chandler TJ, Cumpston J, Li M, Page T, Welch MJ. VA. Cochrane handbook for systematic reviews of interventions version 6.2 (updated February 2021). 2021. www.training.cochrane.org/handbook

[16] Rohatgi A, WebPlotDigitizer, September. 2022; version 4.6]. https://automeris.io/WebPlotDigitizer

[17] Freeman SC, et al. Development of an interactive web-based tool to conduct and interrogate meta-analysis of diagnostic test accuracy studies: MetaDTA. BMC Med Res Methodol. 2019;19(1):81. 30999861 10.1186/s12874-019-0724-x PMC6471890

[18] Hanley JA, McNeil BJ. The meaning and use of the area under a receiver operating characteristic (ROC) curve. Radiology. 1982;143(1):29–36. 7063747 10.1148/radiology.143.1.7063747

[19] Pustejovsky JE, Tipton E. Meta-analysis with robust variance estimation: expanding the range of Working models. Prev Sci. 2022;23(3):425–38. 33961175 10.1007/s11121-021-01246-3

[20] Reitsma JB, et al. Bivariate analysis of sensitivity and specificity produces informative summary measures in diagnostic reviews. J Clin Epidemiol. 2005;58(10):982–90. 16168343 10.1016/j.jclinepi.2005.02.022

[21] Chu H, Cole SR. Bivariate meta-analysis of sensitivity and specificity with sparse data: a generalized linear mixed model approach. J Clin Epidemiol, 2006;59(12):1331-2; author reply 1332-3. 10.1016/j.jclinepi.2006.06.011 17098577

[22] Harbord RM, et al. A unification of models for meta-analysis of diagnostic accuracy studies. Biostatistics. 2007;8(2):239–51. 16698768 10.1093/biostatistics/kxl004

[23] Whiting PF, et al. QUADAS-2: a revised tool for the quality assessment of diagnostic accuracy studies. Ann Intern Med. 2011;155(8):529–36. 22007046 10.7326/0003-4819-155-8-201110180-00009

[24] Abdelaty T, et al. Plasma microRNA-192 expression as a potential biomarker of diabetic kidney disease in patients with type 2 diabetes mellitus. Clin Diabetol. 2020;9(6):454–60. 10.5603/DK.2020.0045

[25] Abdelsalam M, et al. MicroRNA-451 as an early predictor of chronic kidney Disease in Diabetic Nephropathy. Int J Nephrol. 2020;2020:8075376. 32855824 10.1155/2020/8075376 PMC7443237

[26] Abdul-Maksoud RS, et al. Circulating miR-181a and miR-223 expression with the potential value of biomarkers for the diagnosis of systemic lupus erythematosus and predicting lupus nephritis. J Gene Med. 2021;23(5):e3326. 33617143 10.1002/jgm.3326

[27] Abdou AE, et al. Urinary IgG, serum CX3CL1 and miRNA-152-3p: as predictors of nephropathy in Egyptian type 2 diabetic patients. Tissue Barriers. 2022;10(3):1994823. 34689723 10.1080/21688370.2021.1994823 PMC9359404

[28] Akhbari M et al. Expression level of circulating cell free miR-155 gene in serum of patients with Diabetic Nephropathy. Clin Lab, 2019. 65(8). 10.7754/Clin.Lab.2019.190209 31414764

[29] Altamemi IA, Ridha A. Micro-RNA-193a as a focal Segmental Glomerulosclerosis Biomarker. Pharm Sci Res. 2019;11(3):882–5.

[30] M, A., et al., Dysregulated levels of glycogen synthase kinase-3β (GSK-3β) and miR-135 in peripheral blood samples of cases with nephrotic syndrome. PeerJ, 2020. 8e10377). 10.7717/peerj.10377 PMC7749650 33362958

[31] Bai X, et al. Diagnostic value of VDBP and mir-155-5p in diabetic nephropathy and the correlation with urinary microalbumin. Exp Ther Med. 2020;20(5):86. 32968443 10.3892/etm.2020.9214 PMC7500046

[32] Bayomy NR, et al. Mir-142-5p as an indicator of autoimmune processes in childhood idiopathic nephrotic syndrome and as a part of MicroRNAs expression panels for its diagnosis and prediction of response to steroid treatment. Mol Immunol. 2022;141:21–32. 34785326 10.1016/j.molimm.2021.11.004

[33] Beltrami C, et al. Association of elevated urinary miR-126, miR-155, and miR-29b with Diabetic kidney disease. Am J Pathol. 2018;188(9):1982–92. 29981742 10.1016/j.ajpath.2018.06.006

[34] Cardenas-Gonzalez M, et al. Identification, confirmation, and replication of novel urinary MicroRNA biomarkers in Lupus Nephritis and Diabetic Nephropathy. Clin Chem. 2017;63(9):1515–26. 28667184 10.1373/clinchem.2017.274175 PMC5610914

[35] Chen T, et al. Increased urinary exosomal microRNAs in children with idiopathic nephrotic syndrome. EBioMedicine. 2019;39:552–61. 30467011 10.1016/j.ebiom.2018.11.018 PMC6355644

[36] Chun-Yan L, et al. Liquid biopsy biomarkers of renal interstitial fibrosis based on urinary exosome. Exp Mol Pathol. 2018;105(2):223–8. 30121168 10.1016/j.yexmp.2018.08.004

[37] Duan ZY, et al. Selection of urinary sediment miRNAs as specific biomarkers of IgA nephropathy. Sci Rep. 2016;6:23498. 27000966 10.1038/srep23498 PMC4802218

[38] Eissa S, et al. Urinary exosomal microRNA panel unravels novel biomarkers for diagnosis of type 2 diabetic kidney disease. J Diabetes Complications. 2016;30(8):1585–92. 27475263 10.1016/j.jdiacomp.2016.07.012

[39] El-Samahy MH, et al. Urinary miRNA-377 and miRNA-216a as biomarkers of nephropathy and subclinical atherosclerotic risk in pediatric patients with type 1 diabetes. J Diabetes Complications. 2018;32(2):185–92. 29175120 10.1016/j.jdiacomp.2017.10.014

[40] ShereenSaeidElshaer et al. MiR-216a in Diabetic Nephropathy: relation with autophagy and apoptosis. Int J Pharm Res Allied Sci. 7(1): p. 15–24.

[41] Etemadi J, et al. Elevated levels of plasma microRNA-192 in patients with lupus nephritis. Immunopathol Persa. 2019;5(1):e02–02. 10.15171/ipp.2019.02

[42] Feng D, Wu B, Pang Y. Diagnostic value of urinary exosomal miR-23b-3p, miR-30a-5p, and mir-151-3p in children with primary nephrotic syndrome. Transl Androl Urol. 2020;9(5):2235–41. 33209688 10.21037/tau-20-1260 PMC7658171

[43] Fouad M, et al. MicroRNA-21 as an early marker of Nephropathy in patients with type 1 diabetes. Indian J Nephrol. 2020;30(1):21–5. 32015595 10.4103/ijn.IJN_80_19 PMC6977383

[44] Guo S-M et al. Clinical correlation of plasma miR-21, miR-126 and miR-148 a in patients with lupus nephritis. 2016.

[45] Hejazian SM, et al. Expression levels of miR-30c and miR-186 in adult patients with Membranous glomerulonephritis and Focal Segmental Glomerulosclerosis. Int J Nephrol Renovasc Dis. 2020;13:193–201. 32848442 10.2147/IJNRD.S258624 PMC7428378

[46] Hong Y, et al. Plasma miR-193a-3p can be a potential biomarker for the diagnosis of diabetic nephropathy. Ann Clin Biochem. 2021;58(2):141–8. 33302703 10.1177/0004563220983851

[47] Hu H, et al. Circulating MiR-29a, possible use as a Biomarker for Monitoring IgA Nephropathy. Iran J Kidney Dis. 2020;14(2):107–18. 32165595

[48] Huang C, Huang YQ. The correlation of circulating miR-29b and inflammatory markers with albuminuria in hypertensive patients. Clin Exp Hypertens. 2020;42(8):743–7. 32631160 10.1080/10641963.2020.1790585

[49] Huang P, et al. Down-regulation of LINC00667 hinders renal tubular epithelial cell apoptosis and fibrosis through miR-34c. Clin Transl Oncol. 2021;23(3):572–81. 32705492 10.1007/s12094-020-02451-2

[50] Huang YQ, et al. The association of miR-29a with proteinuria in essential hypertension. J Hum Hypertens. 2018;32(11):775–80. 30127486 10.1038/s41371-018-0097-3

[51] Ibrahim AA, et al. Expression of exosomal miR-21 and miR-29 in serum of children and adolescents with T1DM and persistent microalbuminuria. Gene Rep. 2019;16:100461. 10.1016/j.genrep.2019.100461

[52] Ishii H, et al. MicroRNA expression profiling in Diabetic kidney disease. Transl Res. 2021;237:31–52. 34102327 10.1016/j.trsl.2021.05.008

[53] Jia Y, et al. miRNAs in urine extracellular vesicles as predictors of Early-Stage Diabetic Nephropathy. J Diabetes Res. 2016;2016:p7932765. 10.1155/2016/7932765 PMC4749815 26942205

[54] Khoshmirsafa M, et al. Elevated expression of miR-21 and miR-155 in peripheral blood mononuclear cells as potential biomarkers for lupus nephritis. Int J Rheum Dis. 2019;22(3):458–67. 30398001 10.1111/1756-185X.13410

[55] Li H, et al. Changes of miR-155 expression in serum of uremic patients before and after treatment and risk factors analysis. Exp Ther Med. 2020;20(4):3352–60. 32855708 10.3892/etm.2020.9067 PMC7444371

[56] Li J, et al. miR-217 is a useful diagnostic biomarker and regulates human podocyte cells apoptosis via Targeting TNFSF11 in Membranous Nephropathy. Biomed Res Int. 2017;2017:2168767. 29214160 10.1155/2017/2168767 PMC5682891

[57] Li W, et al. Circulating exosomal microRNAs as biomarkers of systemic Lupus Erythematosus. Clin (Sao Paulo). 2020;75:e1528. 10.6061/clinics/2020/e1528 PMC7442402 32876110

[58] Li W, et al. Potential value of urinary exosome-derived let-7c-5p in the diagnosis and progression of type II Diabetic Nephropathy. Clin Lab. 2018;64(5):709–18. 29739042 10.7754/Clin.Lab.2018.171031

[59] Liang S, et al. Urinary sediment miRNAs reflect tubulointerstitial damage and therapeutic response in IgA nephropathy. BMC Nephrol. 2017;18(1):63. 28201996 10.1186/s12882-017-0482-0 PMC5312444

[60] Lin LJ, et al. [Expression and diagnostic value of plasma miR-145 and miR-183 in children with lupus nephritis]. Zhongguo Dang Dai Er Ke Za Zhi. 2020;22(6):632–7. 32571464 10.7499/j.issn.1008-8830.2001013 PMC7390214

[61] Lin M, et al. Dysregulation of miR-638 in diabetic nephropathy and its role in inflammatory response. Diabetol Metab Syndr. 2021;13(1):122. 34715911 10.1186/s13098-021-00744-2 PMC8555262

[62] Liu L, et al. miRNA-483-5p targets HDCA4 to regulate renal tubular damage in Diabetic Nephropathy. Horm Metab Res. 2021;53(8):562–9. 34126643 10.1055/a-1480-7519

[63] Liu L, et al. Detection of microRNA-33a-5p in serum, urine and renal tissue of patients with IgA nephropathy. Exp Ther Med. 2021;21(3):205. 33500698 10.3892/etm.2021.9638 PMC7818539

[64] Luo Y, et al. Increased serum and urinary microRNAs in children with idiopathic nephrotic syndrome. Clin Chem. 2013;59(4):658–66. 23344497 10.1373/clinchem.2012.195297

[65] Lv LL, et al. MicroRNA-29c in urinary exosome/microvesicle as a biomarker of renal fibrosis. Am J Physiol Ren Physiol. 2013;305(8):F1220–7. 10.1152/ajprenal.00148.2013 23946286

[66] Ma N, et al. Circulating microRNA-194 levels in Chinese patients with diabetic kidney disease: a case-control study. Ther Adv Endocrinol Metab. 2021;12:20420188211049615. 34676065 10.1177/20420188211049615 PMC8524709

[67] Martinez-Arroyo O et al. Decreased urinary levels of SIRT1 as non-invasive biomarker of early renal damage in hypertension. Int J Mol Sci, 2020. 21(17). 10.3390/ijms21176390 PMC7503821 32887498

[68] Monjezi A, et al. Resistin, TNF-α, and microRNA 124-3p expressions in peripheral blood mononuclear cells are associated with diabetic nephropathy. Int J Diabetes Developing Ctries. 2022;42(1):62–9. 10.1007/s13410-021-00966-0

[69] Motshwari DD, et al. Novel whole blood MicroRNAs Predicting chronic kidney disease in South africans with Hypertension and Diabetes Mellitus. Appl Sci. 2021;11. 10.3390/app11167674.

[70] Nakhjavani M, et al. Plasma levels of miR-21, miR-150, miR-423 in patients with lupus nephritis. Iran J Kidney Dis. 2019;13(3):198–206. 31209193

[71] Navarro-Quiroz E, et al. High-throughput sequencing reveals circulating miRNAs as potential biomarkers of kidney damage in patients with systemic Lupus Erythematosus. PLoS ONE. 2016;11(11):e0166202. 27835701 10.1371/journal.pone.0166202 PMC5106044

[72] Nossier AI, et al. Determination of certain urinary microRNAs as promising biomarkers in diabetic nephropathy patients using gold nanoparticles. Anal Biochem. 2020;609:113967. 32950495 10.1016/j.ab.2020.113967

[73] Perez-Hernandez J, et al. Increased urinary exosomal MicroRNAs in patients with systemic Lupus Erythematosus. PLoS ONE. 2015;10(9):e0138618. 26390437 10.1371/journal.pone.0138618 PMC4577109

[74] Prabu P, et al. MicroRNAs from urinary extracellular vesicles are non-invasive early biomarkers of diabetic nephropathy in type 2 diabetes patients with the ‘Asian Indian phenotype’. Diabetes Metab. 2019;45(3):276–85. 30165157 10.1016/j.diabet.2018.08.004

[75] Serino G, et al. In a retrospective international study, circulating miR-148b and let-7b were found to be serum markers for detecting primary IgA nephropathy. Kidney Int. 2016;89(3):683–92. 26581012 10.1038/ki.2015.333

[76] Solé C et al. An exosomal urinary miRNA signature for early diagnosis of Renal Fibrosis in Lupus Nephritis. Cells, 2019. 8(8). 10.3390/cells8080773 PMC6721515 31349698

[77] Sui W, et al. Serum microRNAs as new diagnostic biomarkers for pre- and post-kidney transplantation. Transpl Proc. 2014;46(10):3358–62. 10.1016/j.transproceed.2014.08.050 25498051

[78] Sun Y, Wang J, Meng Y. Correlation between polymorphisms of the SIRT1 Gene microRNA Target sites and Diabetic Nephropathy. Genet Test Mol Biomarkers. 2021;25(6):387–98. 34152844 10.1089/gtmb.2020.0261

[79] Szeto CC, et al. Urinary miRNA profile for the diagnosis of IgA nephropathy. BMC Nephrol. 2019;20(1):77. 30832601 10.1186/s12882-019-1267-4 PMC6399975

[80] Tan L, et al. Downregulated serum exosomal miR-451a expression correlates with renal damage and its intercellular communication role in systemic Lupus Erythematosus. Front Immunol. 2021;12:630112. 33643314 10.3389/fimmu.2021.630112 PMC7906989

[81] Tayel SI, et al. Simultaneous Assessment of MicroRNAs 126 and 192 in Diabetic Nephropathy patients and the relation of these MicroRNAs with urinary albumin. Curr Mol Med. 2020;20(5):361–71. 31629394 10.2174/1566524019666191019103918

[82] Zununi Vahed S, et al. Altered levels of immune-regulatory microRNAs in plasma samples of patients with lupus nephritis. Bioimpacts. 2018;8(3):177–83. 30211077 10.15171/bi.2018.20 PMC6128973

[83] Wang J, et al. Expression profiling and clinical significance of plasma MicroRNAs in Diabetic Nephropathy. J Diabetes Res. 2019;2019:5204394. 31218232 10.1155/2019/5204394 PMC6536987

[84] Wang L, et al. Identification of plasma miR-106a-5p and miR-30a-5p as potential biomarkers for mesangial proliferative glomerulonephritis. Clin Biochem. 2020;84:79–86. 32673627 10.1016/j.clinbiochem.2020.07.001

[85] Wang S, Yan X. The value of serum cystatin C combined with MIR-16-5P in the early diagnosis of diabetic kidney disease (DKD). Acta Med Mediterranea. 2020;36:1581–5.

[86] Wang W, et al. Up-regulation of serum MiR-130b-3p level is Associated with renal damage in early Lupus Nephritis. Sci Rep. 2015;5:12644. 26316103 10.1038/srep12644 PMC4551961

[87] Wang Z et al. Let-7c-5p is involved in chronic kidney disease by targeting TGF-β signaling. Biomed Res Int, 2020. 2020: p. 6960941. 10.1155/2020/6960941 PMC7306863 32626757

[88] Wu J, et al. Plasma microRNA signature of patients with IgA nephropathy. Gene. 2018;649:80–6. 29459010 10.1016/j.gene.2018.01.050

[89] Wu L, et al. Altered expression of serum miR-106a, miR-19b, miR-17, and PTEN in patients with idiopathic membranous nephropathy. J Clin Lab Anal. 2021;35(4):e23737. 33745222 10.1002/jcla.23737 PMC8059741

[90] Wu Q, et al. Diagnostic significance of circulating mir-485-5p in patients with lupus nephritis and its predictive value evaluation for the clinical outcomes. J Chin Med Assoc. 2021;84(5):491–7. 33742995 10.1097/JCMA.0000000000000522 PMC12966111

[91] Xiao B, et al. Plasma microRNA panel is a novel biomarker for focal segmental glomerulosclerosis and associated with podocyte apoptosis. Cell Death Dis. 2018;9(5):533. 29748623 10.1038/s41419-018-0569-y PMC5945632

[92] Yang X, et al. Microribonucleic acid-192 as a specific biomarker for the early diagnosis of diabetic kidney disease. J Diabetes Investig. 2017;9(3):602–9. 28940849 10.1111/jdi.12753 PMC5934266

[93] Yavropoulou MP et al. Expression of circulating MicroRNAs linked to bone metabolism in chronic kidney Disease-Mineral and Bone Disorder. Biomedicines, 2020. 8(12). 10.3390/biomedicines8120601 PMC7764659 33322822

[94] Zhang C, et al. Urinary miR-196a predicts disease progression in patients with chronic kidney disease. J Transl Med. 2018;16(1):91. 29636065 10.1186/s12967-018-1470-2 PMC5894160

[95] Zhang H, et al. Cell-related circulating MicroRNAs with the potential value of biomarkers in the Differential diagnosis, and Distinguishment between the Disease Activity and Lupus Nephritis for systemic Lupus Erythematosus. Front Immunol. 2018;9:1473. 30008716 10.3389/fimmu.2018.01473 PMC6033964

[96] Zhang L, et al. Co-expression analysis among microRNAs, long non-coding RNAs, and messenger RNAs to understand the pathogenesis and progression of diabetic kidney disease at the genetic level. Methods. 2017;124:46–56. 28577935 10.1016/j.ymeth.2017.05.023 PMC5540768

[97] Zhang W, Ren Y, Li J. Application of miR-193a/WT1/PODXL axis to estimate risk and prognosis of idiopathic membranous nephropathy. Ren Fail. 2019;41(1):704–17. 31352863 10.1080/0886022X.2019.1642210 PMC6711109

[98] Zhao Y, et al. Urinary exosomal MiRNA-4534 as a Novel Diagnostic Biomarker for Diabetic kidney disease. Front Endocrinol (Lausanne). 2020;11:590. 32982978 10.3389/fendo.2020.00590 PMC7484971

[99] Zhou H, et al. miR-150 promotes renal fibrosis in lupus nephritis by downregulating SOCS1. J Am Soc Nephrol. 2013;24(7):1073–87. 23723424 10.1681/ASN.2012080849 PMC3699828

[100] Zhu Y, Xue Z, Di L. Regulation of MiR-146a and TRAF6 in the diagnose of Lupus Nephritis. Med Sci Monit. 2017;23:2550–7. 28549054 10.12659/MSM.900667 PMC5455804

[101] Li S, et al. Urinary exosomal MicroRNAs as new noninvasive biomarkers of IgA Nephropathy. Tohoku J Exp Med. 2022;256(3):215–23. 35314529 10.1620/tjem.256.215

[102] Habieb MS et al. Evaluation of Mir 29c-3p and Mir 31-5p serum values in Predicting Nephropathy in type 2 Diabetic patients. Am J Biochem Biotechnol, 2022. 18(1).

[103] Szeto CC, et al. Urinary mi-106a for the diagnosis of IgA nephropathy: Liquid biopsy for kidney disease. Clin Chim Acta. 2022;530:81–6. 35278407 10.1016/j.cca.2022.03.006

[104] Pawluczyk IZA, et al. Differential expression of microRNA mir-150-5p in IgA nephropathy as a potential mediator and marker of disease progression. Kidney Int. 2021;99(5):1127–39. 33417998 10.1016/j.kint.2020.12.028

[105] Akpınar K, et al. Mir-21-3p and mir-192-5p in patients with type 2 diabetic nephropathy. Diagnosis (Berl). 2022;9(4):499–507. 35976169 10.1515/dx-2022-0036

[106] Al-Kafaji G, et al. Decreased expression of circulating microRNA-126 in patients with type 2 diabetic nephropathy: a potential blood-based biomarker. Exp Ther Med. 2016;12(2):815–22. 27446281 10.3892/etm.2016.3395 PMC4950785

[107] Li J et al. MicroRNAs as potential biomarkers for the diagnosis of chronic kidney disease: a systematic review and Meta-analysis. Front Med, 2022. 8. 10.3389/fmed.2021.782561 PMC8860181 35198569

[108] Wang C, et al. A panel of five serum miRNAs as a potential diagnostic tool for early-stage renal cell carcinoma. Sci Rep. 2015;5(1):7610. 25556603 10.1038/srep07610 PMC5154588

[109] Bhaskaran V, et al. The functional synergism of microRNA clustering provides therapeutically relevant epigenetic interference in glioblastoma. Nat Commun. 2019;10(1):442. 30683859 10.1038/s41467-019-08390-z PMC6347618

[110] Mahtal N, et al. MicroRNAs in kidney injury and disease. Nat Rev Nephrol. 2022;18(10):643–62. 35974169 10.1038/s41581-022-00608-6

[111] Chung AC, et al. miR-192 mediates TGF-beta/Smad3-driven renal fibrosis. J Am Soc Nephrol. 2010;21(8):1317–25. 20488955 10.1681/ASN.2010020134 PMC2938591

[112] Sun Y, et al. miR-451 suppresses the NF-kappaB-mediated proinflammatory molecules expression through inhibiting LMP7 in diabetic nephropathy. Mol Cell Endocrinol. 2016;433:75–86. 27264074 10.1016/j.mce.2016.06.004

[113] Kölling M, et al. Therapeutic miR-21 silencing ameliorates Diabetic kidney disease in mice. Mol Ther. 2017;25(1):165–80. 28129112 10.1016/j.ymthe.2016.08.001 PMC5363308

[114] Bijkerk R, et al. Hematopoietic microRNA-126 protects against renal ischemia/reperfusion injury by promoting vascular integrity. J Am Soc Nephrol. 2014;25(8):1710–22. 24610930 10.1681/ASN.2013060640 PMC4116053

[115] Condrat CE et al. miRNAs as biomarkers in Disease: latest findings regarding their role in diagnosis and prognosis. Cells, 2020. 9(2). 10.3390/cells9020276 PMC7072450 31979244

[116] Cheng L, et al. Characterization and deep sequencing analysis of exosomal and non-exosomal miRNA in human urine. Kidney Int. 2014;86(2):433–44. 24352158 10.1038/ki.2013.502

[117] Cui C, Cui Q. The relationship of human tissue microRNAs with those from body fluids. Sci Rep. 2020;10(1):5644. 32221351 10.1038/s41598-020-62534-6 PMC7101318

[118] Mall C, et al. Stability of miRNA in human urine supports its biomarker potential. Biomark Med. 2013;7(4):623–31. 23905899 10.2217/bmm.13.44 PMC3885156

[119] Fourdinier O, et al. Serum levels of miR-126 and miR-223 and outcomes in chronic kidney disease patients. Sci Rep. 2019;9(1):4477. 30872798 10.1038/s41598-019-41101-8 PMC6418179

[120] Motshwari DD, et al. Expression of whole blood miR-126-3p, -30a-5p, -1299, -182-5p and – 30e-3p in chronic kidney disease in a South African community-based sample. Sci Rep. 2022;12(1):4107. 35260775 10.1038/s41598-022-08175-3 PMC8904505

[121] Fujii R, et al. Associations of circulating MicroRNAs (miR-17, miR-21, and miR-150) and chronic kidney disease in a Japanese Population. J Epidemiol. 2020;30(4):177–82. 30905898 10.2188/jea.JE20180233 PMC7064557

[122] Du S, et al. Applying serum proteins and MicroRNA as novel biomarkers for early-stage cervical Cancer detection. Sci Rep. 2020;10(1):9033. 32493989 10.1038/s41598-020-65850-z PMC7271168

[123] Hegyi P, et al. Accelerating the translational medicine cycle: the Academia Europaea pilot. Nat Med. 2021;27(8):1317–9. 34312557 10.1038/s41591-021-01458-8

