# Supplementary material for: Panel miRNAs are potential diagnostic markers for chronic kidney diseases: a systematic review and meta-analysis
Source: BMC Nephrol. 2024 Aug 13;25:261. doi: 10.1186/s12882-024-03702-y (PMC11323638; doi:10.1186/s12882-024-03702-y)
Supplement: Supplementary file 1 — Supplementary Material 1 [file 12882_2024_3702_MOESM1_ESM.docx]

# **Supplementary File**

**Panel miRNAs are potential diagnostic markers for chronic kidney diseases: a systematic review and meta-analysis**

Gantsetseg Garmaa^1,2,3^, Rita Nagy^2^, Tamás Kói^2,5^, Uyen Nguyen Do To^2,6^, Dorottya Gergő^2,7^, Dénes Kleiner^2,8^, Dezső Csupor^2,9,10^, Péter Hegyi^2,9,11^, Gábor Kökény^1,12^

**Affiliations**

^1^ Institute of Translational Medicine, Semmelweis University, 1089 Budapest, Nagyvárad tér 4, Hungary

^2^ Center for Translational Medicine, Semmelweis University, 1085 Budapest, Üllői út 26, Hungary

^3^ Department of Pathology, School of Medicine, Mongolian National University of Medical Sciences, Ulan-Bator, 14210, Mongolia

^4^ Heim Pál National Pediatric Institute, 1089 Budapest, Üllői út 86, Hungary

^5^ Department of Stochastics, Institute of Mathematics, Budapest University of Technology and Economics, Budapest, Hungary

^6^ András Pető Faculty, Semmelweis University, 1085 Budapest, Üllői út 26, Hungary

^7^ Department of Pharmacognosy, Semmelweis University, 1085 Budapest, Üllői út 26. Hungary

^8^ University Pharmacy Department of Pharmacy Administration, Semmelweis University, Budapest, Hungary

^9^ Institute for Translational Medicine, Medical School, University of Pécs, 7624 Pécs, Szigeti út 12, Hungary

^10^ Institute of Clinical Pharmacy, University of Szeged, 6725 Szeged, Szikra utca 8, Hungary

^11^ Institute of Pancreatic Diseases, Semmelweis University, Tömő street 25-29, Budapest, Hungary

^12^ International Nephrology Research and Training Center, Semmelweis University, 1089 Budapest, Nagyvárad tér 4, Hungary

# SUPPLEMENTARY METHODS

## Search strategy

**Date:** November 25^th^, 2021 and November 26^th^, 2022

**Search key principle:**

| **Databases** | **Search Key** |
| --- | --- |
| Cochrane Central Register of Controlled Trials (CENTRAL) | (micro RNA OR microRNA or microRNAs OR miRNA or miRNAs OR miRs) AND (diabetic nephropathies OR diabetic nephropathy OR chronic kidney disease OR chronic kidney failure or chronic kidney insufficiency or chronic renal failure OR renal disease or chronic renal insufficiency or chronic renal disease OR "kidney fibrosis" OR "renal fibrosis" OR "renal interstitial A  fibrosis" OR nephropathy OR nephropat* OR nephritis OR nephrit* OR glomerul* OR "Kimmelstiel-Wilson") |
| Web of Science | (micro RNA OR microRNA or microRNAs OR miRNA or miRNAs OR miRs) AND (diabetic nephropathies OR diabetic nephropathy OR chronic kidney disease OR chronic kidney failure or chronic kidney insufficiency or chronic renal failure OR renal disease or chronic renal insufficiency or chronic renal disease OR "kidney fibrosis" OR "renal fibrosis" OR "renal interstitial fibrosis" OR nephropathy OR nephropat* OR nephritis OR nephrit* OR glomerul* OR "Kimmelstiel-Wilson") |
| Embase | (micro RNA OR microRNA or microRNAs OR miRNA or miRNAs OR miRs) AND (diabetic nephropathies OR diabetic nephropathy OR chronic kidney disease OR chronic kidney failure or chronic kidney insufficiency or chronic renal failure OR renal disease or chronic renal insufficiency or chronic renal disease OR "kidney fibrosis" OR "renal fibrosis" OR "renal interstitial fibrosis" OR nephropathy OR nephropat* OR nephritis OR nephrit* OR glomerul* OR "Kimmelstiel-Wilson") |
| MEDLINE (via NCBI PubMed) | (micro RNA OR microRNA or microRNAs OR miRNA or miRNAs OR miRs) AND (diabetic nephropathies OR diabetic nephropathy OR chronic kidney disease OR chronic kidney failure or chronic kidney insufficiency or chronic renal failure OR renal disease or chronic renal insufficiency or chronic renal disease OR "kidney fibrosis" OR "renal fibrosis" OR "renal interstitial fibrosis" OR nephropathy OR nephropat* OR nephritis OR nephrit* OR glomerul* OR "Kimmelstiel-Wilson") |
| Scopus | (micro RNA OR microRNA or microRNAs OR miRNA or miRNAs OR miRs) AND (diabetic nephropathies OR diabetic nephropathy OR chronic kidney disease OR chronic kidney failure or chronic kidney insufficiency or chronic renal failure OR renal disease or chronic renal insufficiency or chronic renal disease OR "kidney fibrosis" OR "renal fibrosis" OR "renal interstitial fibrosis" OR nephropathy OR nephropat* OR nephritis OR nephrit* OR glomerul* OR "Kimmelstiel-Wilson") |

**Search key and MeSH terms:**

("micrornas"[MeSH Terms] OR "micrornas"[All Fields] OR ("micro"[All Fields] AND "rna"[All Fields]) OR "micro rna"[All Fields] OR ("microrna s"[All Fields] OR "micrornas"[MeSH Terms] OR "micrornas"[All Fields] OR "microrna"[All Fields]) OR ("microrna s"[All Fields] OR "micrornas"[MeSH Terms] OR "micrornas"[All Fields] OR "microrna"[All Fields]) OR ("micrornas"[MeSH Terms] OR "micrornas"[All Fields] OR "mirna"[All Fields] OR "mirnas"[All Fields] OR "mirna s"[All Fields]) OR ("micrornas"[MeSH Terms] OR "micrornas"[All Fields] OR "mirna"[All Fields] OR "mirnas"[All Fields] OR "mirna s"[All Fields]) OR "miRs"[All Fields]) AND ("diabetic nephropathies"[MeSH Terms] OR ("diabetic"[All Fields] AND "nephropathies"[All Fields]) OR "diabetic nephropathies"[All Fields] OR ("diabetic nephropathies"[MeSH Terms] OR ("diabetic"[All Fields] AND "nephropathies"[All Fields]) OR "diabetic nephropathies"[All Fields] OR ("diabetic"[All Fields] AND "nephropathy"[All Fields]) OR "diabetic nephropathy"[All Fields]) OR ("renal insufficiency, chronic"[MeSH Terms] OR ("renal"[All Fields] AND "insufficiency"[All Fields] AND "chronic"[All Fields]) OR "chronic renal insufficiency"[All Fields] OR ("chronic"[All Fields] AND "kidney"[All Fields] AND "disease"[All Fields]) OR "chronic kidney disease"[All Fields]) OR ("kidney failure, chronic"[MeSH Terms] OR ("kidney"[All Fields] AND "failure"[All Fields] AND "chronic"[All Fields]) OR "chronic kidney failure"[All Fields] OR ("chronic"[All Fields] AND "kidney"[All Fields] AND "failure"[All Fields])) OR ("renal insufficiency, chronic"[MeSH Terms] OR ("renal"[All Fields] AND "insufficiency"[All Fields] AND "chronic"[All Fields]) OR "chronic renal insufficiency"[All Fields] OR ("chronic"[All Fields] AND "kidney"[All Fields] AND "insufficiency"[All Fields]) OR "chronic kidney insufficiency"[All Fields]) OR ("kidney failure, chronic"[MeSH Terms] OR ("kidney"[All Fields] AND "failure"[All Fields] AND "chronic"[All Fields]) OR "chronic kidney failure"[All Fields] OR ("chronic"[All Fields] AND "renal"[All Fields] AND "failure"[All Fields]) OR "chronic renal failure"[All Fields]) OR ("kidney diseases"[MeSH Terms] OR ("kidney"[All Fields] AND "diseases"[All Fields]) OR "kidney diseases"[All Fields] OR ("renal"[All Fields] AND "disease"[All Fields]) OR "renal disease"[All Fields]) OR ("renal insufficiency, chronic"[MeSH Terms] OR ("renal"[All Fields] AND "insufficiency"[All Fields] AND "chronic"[All Fields]) OR "chronic renal insufficiency"[All Fields] OR ("chronic"[All Fields] AND "renal"[All Fields] AND "insufficiency"[All Fields])) OR ("renal insufficiency, chronic"[MeSH Terms] OR ("renal"[All Fields] AND "insufficiency"[All Fields] AND "chronic"[All Fields]) OR "chronic renal insufficiency"[All Fields] OR ("chronic"[All Fields] AND "renal"[All Fields] AND "disease"[All Fields]) OR "chronic renal disease"[All Fields] OR "kidney failure, chronic"[MeSH Terms] OR ("kidney"[All Fields] AND "failure"[All Fields] AND "chronic"[All Fields]) OR "chronic kidney failure"[All Fields] OR ("chronic"[All Fields] AND "renal"[All Fields] AND "disease"[All Fields])) OR "kidney fibrosis"[All Fields] OR "renal fibrosis"[All Fields] OR "renal interstitial fibrosis"[All Fields] OR ("kidney diseases"[MeSH Terms] OR ("kidney"[All Fields] AND "diseases"[All Fields]) OR "kidney diseases"[All Fields] OR "nephropathies"[All Fields] OR "nephropathy"[All Fields]) OR "nephropat*"[All Fields] OR ("nephritis"[MeSH Terms] OR "nephritis"[All Fields] OR "nephritides"[All Fields]) OR "nephrit*"[All Fields] OR "glomerul*"[All Fields] OR "Kimmelstiel-Wilson"[All Fields])

**Translations for search key:**

**micro RNA:** "micrornas"[MeSH Terms] OR "micrornas"[All Fields] OR ("micro"[All Fields] AND "rna"[All Fields]) OR "micro rna"[All Fields]

**microRNA:** "microrna's"[All Fields] OR "micrornas"[MeSH Terms] OR "micrornas"[All Fields] OR "microrna"[All Fields]

**microRNAs**: "microrna's"[All Fields] OR "micrornas"[MeSH Terms] OR "micrornas"[All Fields] OR "microrna"[All Fields]

**miRNA:** "micrornas"[MeSH Terms] OR "micrornas"[All Fields] OR "mirna"[All Fields] OR "mirnas"[All Fields] OR "mirna's"[All Fields]

**miRNAs:** "micrornas"[MeSH Terms] OR "micrornas"[All Fields] OR "mirna"[All Fields] OR "mirnas"[All Fields] OR "mirna's"[All Fields]

**diabetic nephropathies:** "diabetic nephropathies"[MeSH Terms] OR ("diabetic"[All Fields] AND "nephropathies"[All Fields]) OR "diabetic nephropathies"[All Fields]

**diabetic nephropathy:** "diabetic nephropathies"[MeSH Terms] OR ("diabetic"[All Fields] AND "nephropathies"[All Fields]) OR "diabetic nephropathies"[All Fields] OR ("diabetic"[All Fields] AND "nephropathy"[All Fields]) OR "diabetic nephropathy"[All Fields]

**chronic kidney disease:** "renal insufficiency, chronic"[MeSH Terms] OR ("renal"[All Fields] AND "insufficiency"[All Fields] AND "chronic"[All Fields]) OR "chronic renal insufficiency"[All Fields] OR ("chronic"[All Fields] AND "kidney"[All Fields] AND "disease"[All Fields]) OR "chronic kidney disease"[All Fields]

**chronic kidney failure:** "kidney failure, chronic"[MeSH Terms] OR ("kidney"[All Fields] AND "failure"[All Fields] AND "chronic"[All Fields]) OR "chronic kidney failure"[All Fields] OR ("chronic"[All Fields] AND "kidney"[All Fields] AND "failure"[All Fields])

**chronic kidney insufficiency:** "renal insufficiency, chronic"[MeSH Terms] OR ("renal"[All Fields] AND "insufficiency"[All Fields] AND "chronic"[All Fields]) OR "chronic renal insufficiency"[All Fields] OR ("chronic"[All Fields] AND "kidney"[All Fields] AND "insufficiency"[All Fields]) OR "chronic kidney insufficiency"[All Fields]

**chronic renal failure:** "kidney failure, chronic"[MeSH Terms] OR ("kidney"[All Fields] AND "failure"[All Fields] AND "chronic"[All Fields]) OR "chronic kidney failure"[All Fields] OR ("chronic"[All Fields] AND "renal"[All Fields] AND "failure"[All Fields]) OR "chronic renal failure"[All Fields]

**renal disease:** "kidney diseases"[MeSH Terms] OR ("kidney"[All Fields] AND "diseases"[All Fields]) OR "kidney diseases"[All Fields] OR ("renal"[All Fields] AND "disease"[All Fields]) OR "renal disease"[All Fields]

**chronic renal insufficiency:** "renal insufficiency, chronic"[MeSH Terms] OR ("renal"[All Fields] AND "insufficiency"[All Fields] AND "chronic"[All Fields]) OR "chronic renal insufficiency"[All Fields] OR ("chronic"[All Fields] AND "renal"[All Fields] AND "insufficiency"[All Fields])

**chronic renal disease:** "renal insufficiency, chronic"[MeSH Terms] OR ("renal"[All Fields] AND "insufficiency"[All Fields] AND "chronic"[All Fields]) OR "chronic renal insufficiency"[All Fields] OR ("chronic"[All Fields] AND "renal"[All Fields] AND "disease"[All Fields]) OR "chronic renal disease"[All Fields] OR "kidney failure, chronic"[MeSH Terms] OR ("kidney"[All Fields] AND "failure"[All Fields] AND "chronic"[All Fields]) OR "chronic kidney failure"[All Fields] OR ("chronic"[All Fields] AND "renal"[All Fields] AND "disease"[All Fields])

**nephropathy:** "kidney diseases"[MeSH Terms] OR ("kidney"[All Fields] AND "diseases"[All Fields]) OR "kidney diseases"[All Fields] OR "nephropathies"[All Fields] OR "nephropathy"[All Fields]

**nephritis:** "nephritis"[MeSH Terms] OR "nephritis"[All Fields] OR "nephritides"[All Fields]

## Statistical method

Statistical analyses were carried out using the R statistical software (version 4.1.2.) and the R script of the online tool described previously [1]. A p-value of less than 0.05 was considered significant for all statistical analyses.

We collected AUC values and their confidence intervals. We estimated the standard deviations of the AUC values from the confidence intervals. When a confidence interval was unavailable, we used the published formula [2].

The main statistical challenge is that AUC values within a study corresponding to different miRNA-s typically belong to the same population. To create statistically rigorous pooled AUC values (pAUC), we had to consider both the correlations between the sample errors and the correlations between the random effects corresponding to the outcomes present in the same study. To account for these correlations, we fitted multivariate mixed effect models using the rma.mv() function of the metafor R package. We used a hierarchical random effect correlation structure. To circumvent the problem caused by the unknown correlations between the sample errors, we supplemented the method with the robust approach of Pustejovsky et al. [3] implemented in the coef_test() function of the clubSandwhich R package. As an initial guess, we assumed the same correlation (0.6) between any two miRNA AUC corresponding to the same sample, and then we applied the robust correction of the clubSandwhich package.

First, we performed univariate subgroup analyses with the rma.mv() functions. We only put one variable in the model. We visualized the pooled results the individual study AUC values with CI-s on forest plots. We also performed multivariate analysis where we put all the covariates in the fixed effect part of the rma.mv() running mentioned in the previous paragraph.

We highlight that we were able to perform quite a lot miRNA specific AUC meta-analyses. The overall AUC pool is excellent for investigating the overall effects of the variables. However, only the miRNA-specific pAUC has real clinical significance.

Besides AUC values, two by two contingency tables were extracted/calculated from the studies containing the true positive, false positive, false negative, and true negative values. In some cases, we had to use webplotdigitizer available at https://apps.automeris.io/wpd/ to extract sensitivity and specificity values from which we were able to calculate the entries of the contingency table.

In the area of diagnostic meta-analysis one approach is to calculate summary ROC curve for example by following Rutter and Gatsonis et.al [4] . The alternative is the bivariate model of [5, 6] that focuses on pooled sensitivity and specificity. Interestingly, it was shown in Harbord et al. [7] that the two approaches are mathematically equivalent, only the focuses are different. Since our data belongs to many different mRNA-s and, in most cases, we do not have any information about the employed cut-off, we decided to follow the bivariate model [5, 6]. Note, however, that due to the mentioned heterogeneity of the mRNA-s and the cut-off usages, the clinical relevance of the pooled results is limited. Also, the previously mentioned population-related correlations are an extra difficulty in this case. In this case, we treated this difficulty with random selection: we randomly chose only one miRNA from each study and then calculated pooled sensitivity and specificity on the selected subsample. We plotted on ROC plot the sensitivities and specificities of the included studies, the summary estimates of sensitivity and specificity, and the corresponding 95% confidence and prediction regions. In these visualizations, the sizes of the ellipsoids reflect the weights of the studies calculated according to the method described previously [8]. The size of the prediction region gives a glimpse into the mentioned heterogeneity. As a sensitivity analysis, we repeated this random procedure several times. We highlight that we were able to analyze the diabetic nephropathy (DN) prediction performance of miRNA mir-192 separately.

Due to the complicatedness of the applied method, the statistical analysis of heterogeneity is problematic. Moreover, the forest plots revealed that the heterogeneity is relatively high. For these reasons, besides the separate pools of certain frequent mRNA-s (in these cases, we calculated classical I^2^ statistics), we omitted the formal heterogeneity analyses.

We performed the publication bias analyses of AUC values by random selection. First, we created classical funnel plots containing all the rows of the data table. Then we randomly selected one result from each study, and we performed Egger’s test on the chosen dataset. We repeated the random choice several times.

## Risk of bias assessment:

The Quality Assessment of Diagnostic Accuracy Studies (QUADAS-2) guideline – definition of domains:

1. **PATIENT SELECTION**

**Risk of bias - Could the selection of patients have introduced bias?**

| Yes: risk of bias is low | No: potential for bias exists | Unclear: only when insufficient data are reported |
| --- | --- | --- |

**Applicability - Is there a concern that the included patients do not match the review question?**

| Yes: risk of bias is low | No: potential for bias exists | Unclear: only when insufficient data are reported |
| --- | --- | --- |

1. **INDEX TEST**

**Risk of bias - Could the conduct or interpretation of the index test have introduced bias?**

| Yes: risk of bias is low | No: potential for bias exists | Unclear: only when insufficient data are reported |
| --- | --- | --- |

**Applicability - Is there concern that the index test, its conduct, or its interpretation differs from the review question?**

| Yes: risk of bias is low | No: potential for bias exists | Unclear: only when insufficient data are reported |
| --- | --- | --- |

1. **REFERENCE STANDARD**

**Risk of bias - Could the reference standard, conduct, or interpretation have introduced bias?**

| Yes: risk of bias is low | No: potential for bias exists | Unclear: only when insufficient data are reported |
| --- | --- | --- |

**Applicability - Is there a concern that the target condition, defined by the reference standard, does not match the review question?**

| Yes: risk of bias is low | No: potential for bias exists | Unclear: only when insufficient data are reported |
| --- | --- | --- |

1. **FLOW AND TIMING**

**Risk of bias - Could the patient flow have introduced bias?**

| Yes: risk of bias is low | No: potential for bias exists | Unclear: only when insufficient data are reported |
| --- | --- | --- |

## Supplementary Table S1. PRISMA 2020 Checklist

| **Section and Topic** | **Item #** | **Checklist item** | **Location where item is reported** |
| --- | --- | --- | --- |
| **TITLE** | | |  |
| Title | 1 | Identify the report as a systematic review. | 1 |
| **ABSTRACT** | | |  |
| Abstract | 2 | See the PRISMA 2020 for Abstracts checklist. | 3 |
| **INTRODUCTION** | | |  |
| Rationale | 3 | Describe the rationale for the review in the context of existing knowledge. | 5 |
| Objectives | 4 | Provide an explicit statement of the objective(s) or question(s) the review addresses. | 5 |
| **METHODS** | | |  |
| Eligibility criteria | 5 | Specify the inclusion and exclusion criteria for the review and how studies were grouped for the syntheses. | 6 |
| Information sources | 6 | Specify all databases, registers, websites, organisations, reference lists and other sources searched or consulted to identify studies. Specify the date when each source was last searched or consulted. | 6 |
| Search strategy | 7 | Present the full search strategies for all databases, registers and websites, including any filters and limits used. | Supplementary method |
| Selection process | 8 | Specify the methods used to decide whether a study met the inclusion criteria of the review, including how many reviewers screened each record and each report retrieved, whether they worked independently, and if applicable, details of automation tools used in the process. | 6-7 |
| Data collection process | 9 | Specify the methods used to collect data from reports, including how many reviewers collected data from each report, whether they worked independently, any processes for obtaining or confirming data from study investigators, and if applicable, details of automation tools used in the process. | 7 |
| Data items | 10a | List and define all outcomes for which data were sought. Specify whether all results that were compatible with each outcome domain in each study were sought (e.g. for all measures, time points, analyses), and if not, the methods used to decide which results to collect. | 7-8 |
|  | 10b | List and define all other variables for which data were sought (e.g. participant and intervention characteristics, funding sources). Describe any assumptions made about any missing or unclear information. | 7-8 |
| Study risk of bias assessment | 11 | Specify the methods used to assess risk of bias in the included studies, including details of the tool(s) used, how many reviewers assessed each study and whether they worked independently, and if applicable, details of automation tools used in the process. | 8 |
| Effect measures | 12 | Specify for each outcome the effect measure(s) (e.g. risk ratio, mean difference) used in the synthesis or presentation of results. | 7-8 |
| Synthesis methods | 13a | Describe the processes used to decide which studies were eligible for each synthesis (e.g. tabulating the study intervention characteristics and comparing against the planned groups for each synthesis (item #5)). | 7-8 |
|  | 13b | Describe any methods required to prepare the data for presentation or synthesis, such as handling of missing summary statistics, or data conversions. | 7-8 |
|  | 13c | Describe any methods used to tabulate or visually display results of individual studies and syntheses. | 7-8 |
|  | 13d | Describe any methods used to synthesize results and provide a rationale for the choice(s). If meta-analysis was performed, describe the model(s), method(s) to identify the presence and extent of statistical heterogeneity, and software package(s) used. | 7-8 |
|  | 13e | Describe any methods used to explore possible causes of heterogeneity among study results (e.g. subgroup analysis, meta-regression). | 7-8 |
|  | 13f | Describe any sensitivity analyses conducted to assess robustness of the synthesized results. | 7-8 |
| Reporting bias assessment | 14 | Describe any methods used to assess risk of bias due to missing results in a synthesis (arising from reporting biases). | 10 |
| Certainty assessment | 15 | Describe any methods used to assess certainty (or confidence) in the body of evidence for an outcome. | NA |
| **RESULTS** | | |  |
| Study selection | 16a | Describe the results of the search and selection process, from the number of records identified in the search to the number of studies included in the review, ideally using a flow diagram. | 8 |
|  | 16b | Cite studies that might appear to meet the inclusion criteria, but which were excluded, and explain why they were excluded. | NA |
| Study characteristics | 17 | Cite each included study and present its characteristics. | 8 |
| Risk of bias in studies | 18 | Present assessments of risk of bias for each included study. | Supplementary results |
| Results of individual studies | 19 | For all outcomes, present, for each study: (a) summary statistics for each group (where appropriate) and (b) an effect estimate and its precision (e.g. confidence/credible interval), ideally using structured tables or plots. | 8-9 |
| Results of syntheses | 20a | For each synthesis, briefly summarise the characteristics and risk of bias among contributing studies. | 8-9 |
|  | 20b | Present results of all statistical syntheses conducted. If meta-analysis was done, present for each the summary estimate and its precision (e.g. confidence/credible interval) and measures of statistical heterogeneity. If comparing groups, describe the direction of the effect. | 8-9 |
|  | 20c | Present results of all investigations of possible causes of heterogeneity among study results. | 10 |
|  | 20d | Present results of all sensitivity analyses conducted to assess the robustness of the synthesized results. | 10 |
| Reporting biases | 21 | Present assessments of risk of bias due to missing results (arising from reporting biases) for each synthesis assessed. | Supplementary results |
| Certainty of evidence | 22 | Present assessments of certainty (or confidence) in the body of evidence for each outcome assessed. | NA |
| **DISCUSSION** | | |  |
| Discussion | 23a | Provide a general interpretation of the results in the context of other evidence. | 11-12 |
|  | 23b | Discuss any limitations of the evidence included in the review. | 11-12 |
|  | 23c | Discuss any limitations of the review processes used. | 12 |
|  | 23d | Discuss implications of the results for practice, policy, and future research. | 13 |
| **OTHER INFORMATION** | | |  |
| Registration and protocol | 24a | Provide registration information for the review, including register name and registration number, or state that the review was not registered. | 6 |
|  | 24b | Indicate where the review protocol can be accessed, or state that a protocol was not prepared. | 6 |
|  | 24c | Describe and explain any amendments to information provided at registration or in the protocol. | NA |
| Support | 25 | Describe sources of financial or non-financial support for the review, and the role of the funders or sponsors in the review. | 14 |
| Competing interests | 26 | Declare any competing interests of review authors. | 14 |
| Availability of data, code and other materials | 27 | Report which of the following are publicly available and where they can be found: template data collection forms; data extracted from included studies; data used for all analyses; analytic code; any other materials used in the review. | 15 |

*From:*  Page MJ, McKenzie JE, Bossuyt PM, Boutron I, Hoffmann TC, Mulrow CD, et al. The PRISMA 2020 statement: an updated guideline for reporting systematic reviews. BMJ 2021;372:n71. doi: 10.1136/bmj.n71

For more information, visit: <http://www.prisma-statement.org/>

## SUPPLEMENTARY RESULTS

### Supplementary Figure S1. Prisma flow chart - study selection process.


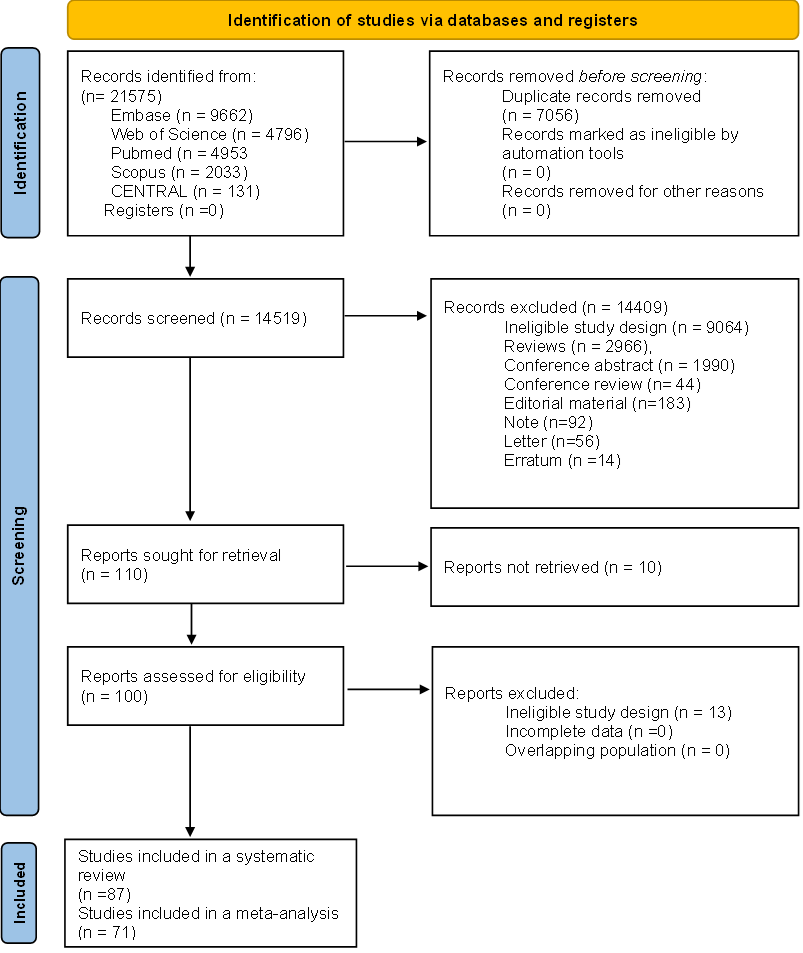


### Supplementary Figure S2. The overall AUC value of miRNAs in kidney disease grouped by healthy and people with chronic diseases.


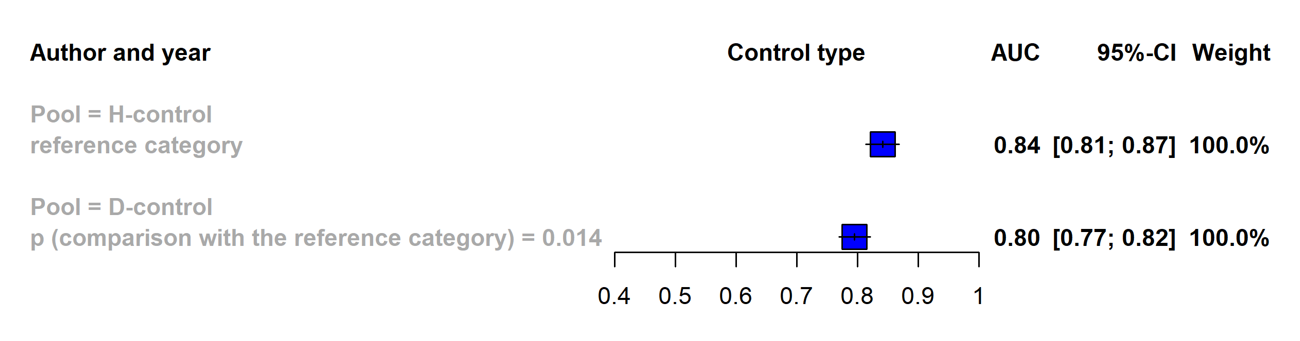


Legend: The overall AUC value of miRNAs in kidney disease are shown by control types; A. healthy and B. chronic disease groups.

### Supplementary Figure S3. The overall AUC value of miRNAs in kidney disease grouped by single and panel miRNAs; A. healthy and B. people with chronic diseases.

**A.**


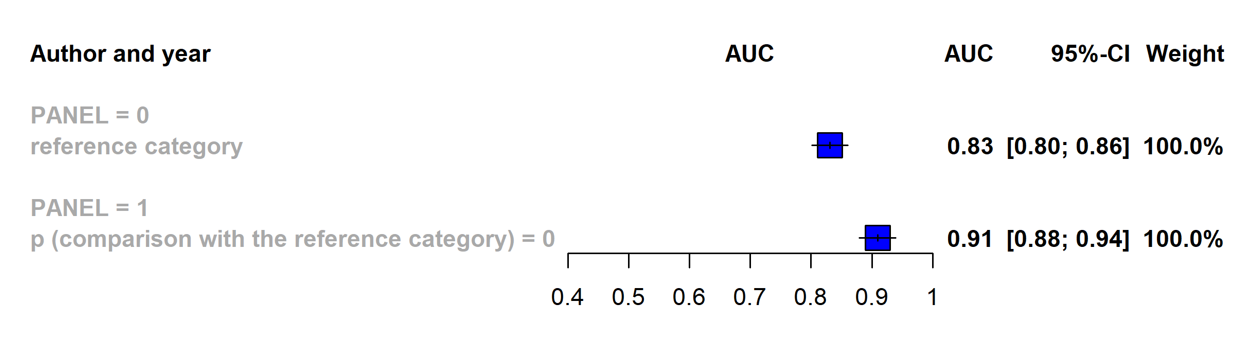


**B.**

**
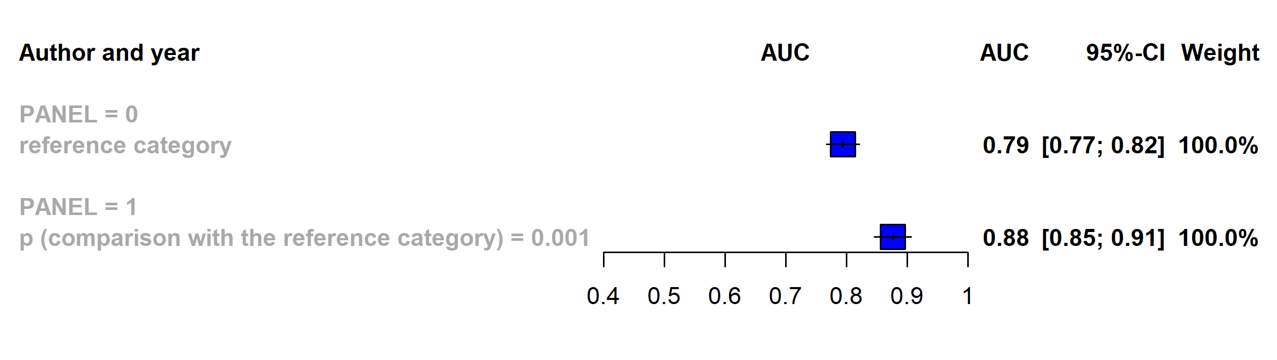
**

Legend: The overall AUC values of single and panel miRNAs are shown. A. healthy and B. chronic disease groups.

### Supplementary Figure S4. Univariate analysis of AUC values in kidney diseases grouped by different sample types; A. healthy control and B. chronic disease groups.

**A.**


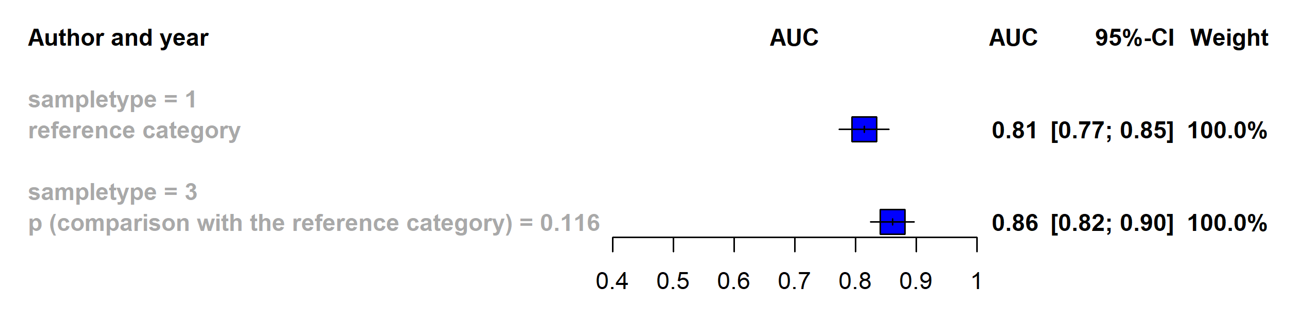


**B.**


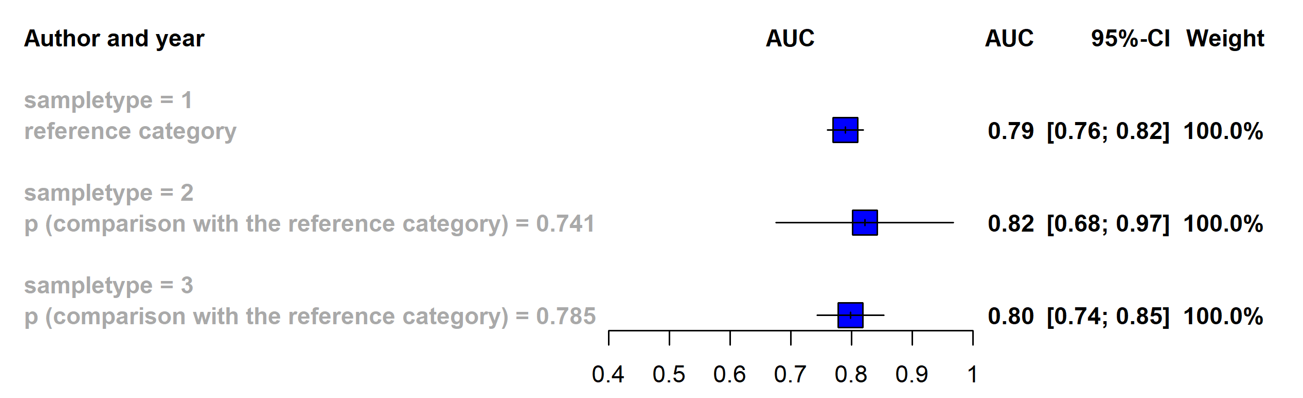


Legend: The overall AUC values of miRNAs in CKD are shown by different samples; 1 - blood, 2 – kidney biopsy, 3 -urine and control types; A. healthy and B. chronic disease groups.

### Supplementary Figure S5. Univariate analysis of single miRNA AUC values in kidney diseases; A. healthy and B. chronic disease groups.

**A.**


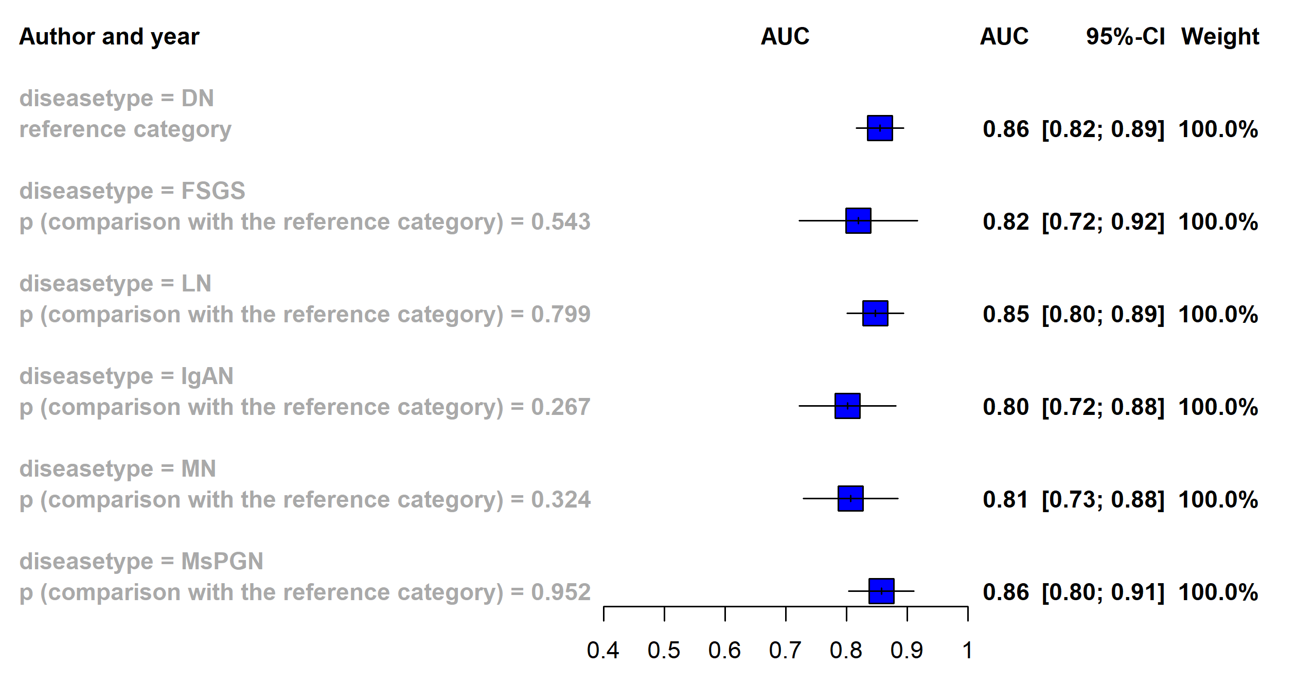


**B.**


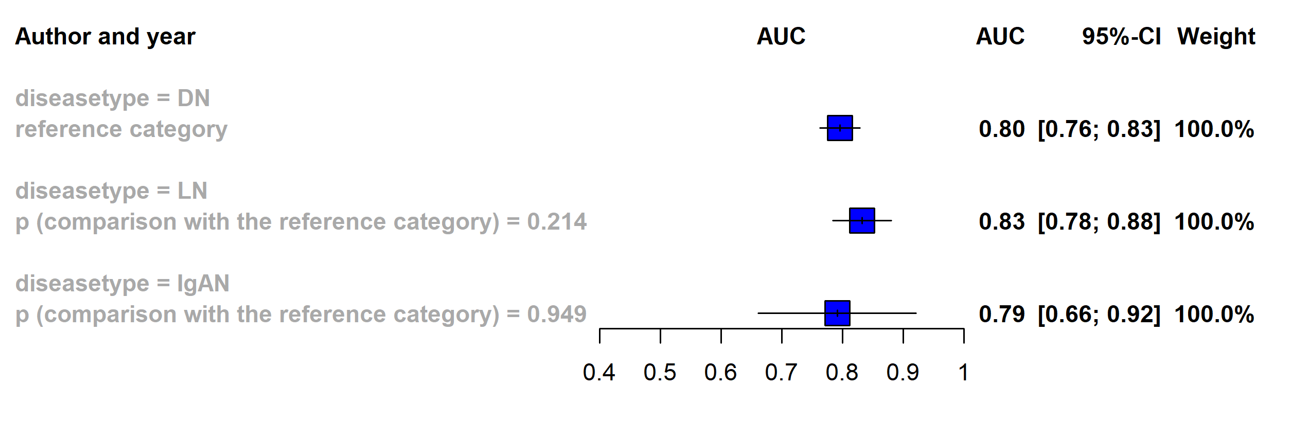


Legend: The overall AUC values of miRNAs in different CKDs are shown by control types; A. healthy and B. chronic disease groups – abbreviation; DN - diabetic nephropathy, FSGS – focal segmental glomerulosclerosis, LN – lupus nephritis, IgAN – IgA nephropathy, MN - membranous nephropathy, MsPGN - Mesangial proliferative glomerulonephritis.

### Supplementary Figure S6. The overall AUC value of miRNAs in kidney diseases grouped by common ethnicities, A. healthy and B. chronic disease groups.

A.


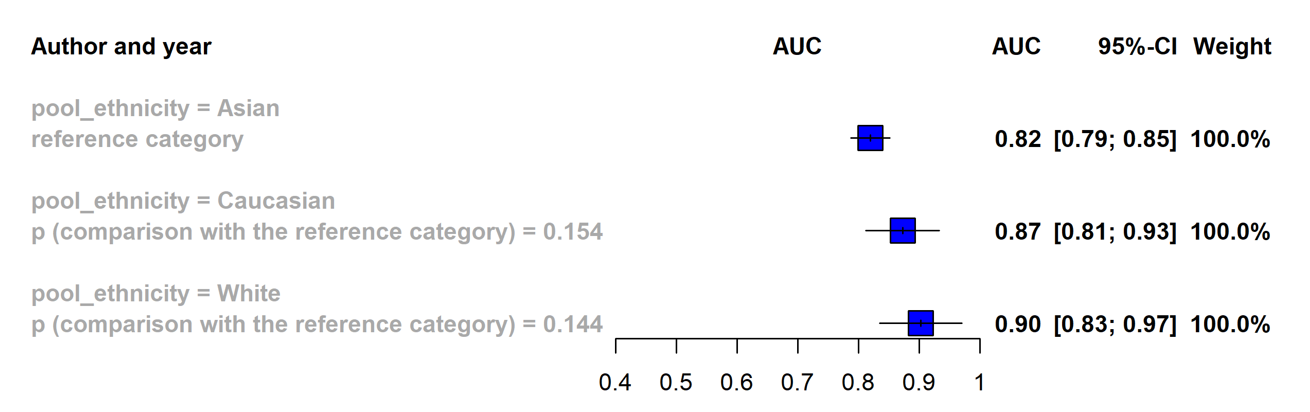


B.


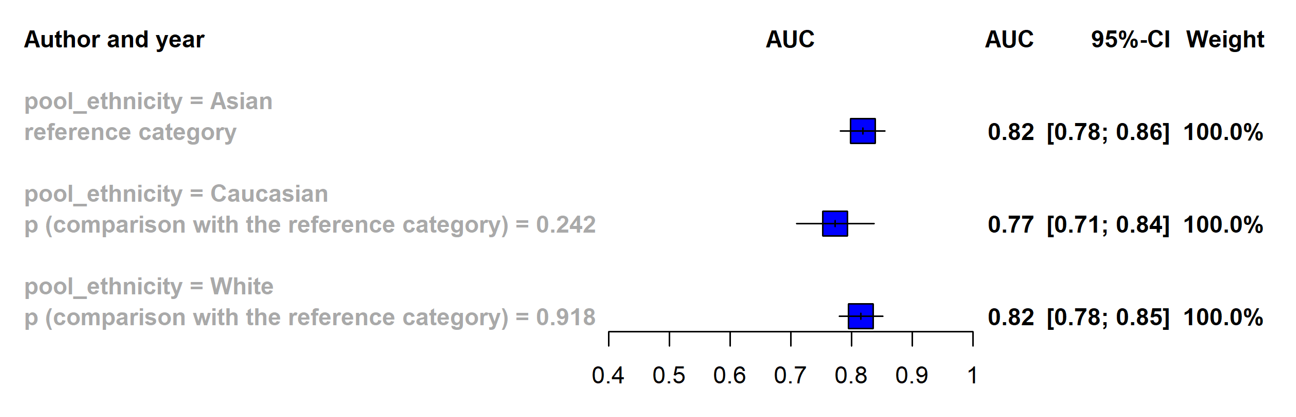


Legend: The overall AUC values of miRNAs in CKD are shown by most reported ethnicities.

### Supplementary Figure S7. The pooled AUC values of miR-146 in CKD comparison with A. healthy controls and, B. chronic disease groups.

**A.**


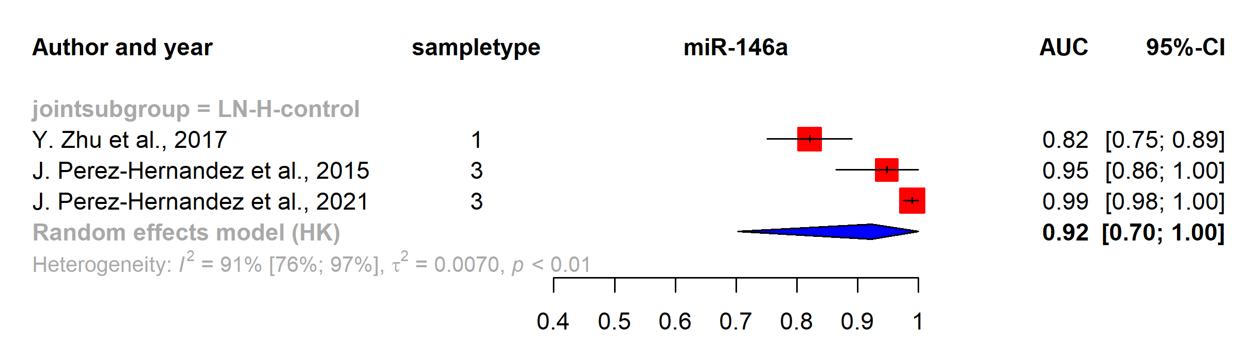


**B.**


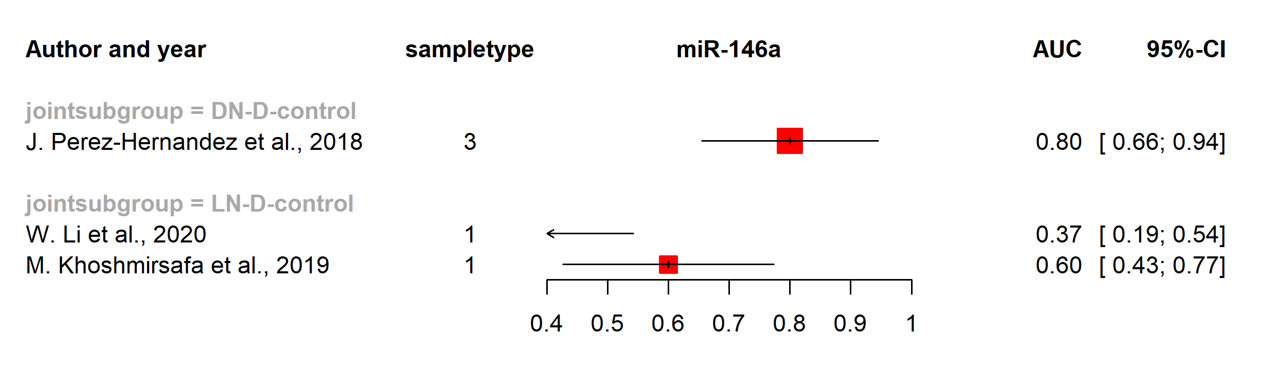


Legend: The overall AUC values of miR-146a in CKD are shown by different control types; A. healthy and B. chronic disease groups -abbreviation; H – healthy, D – diseased.

### Supplementary Figure S8. The publication bias for DN vs. DM studies.


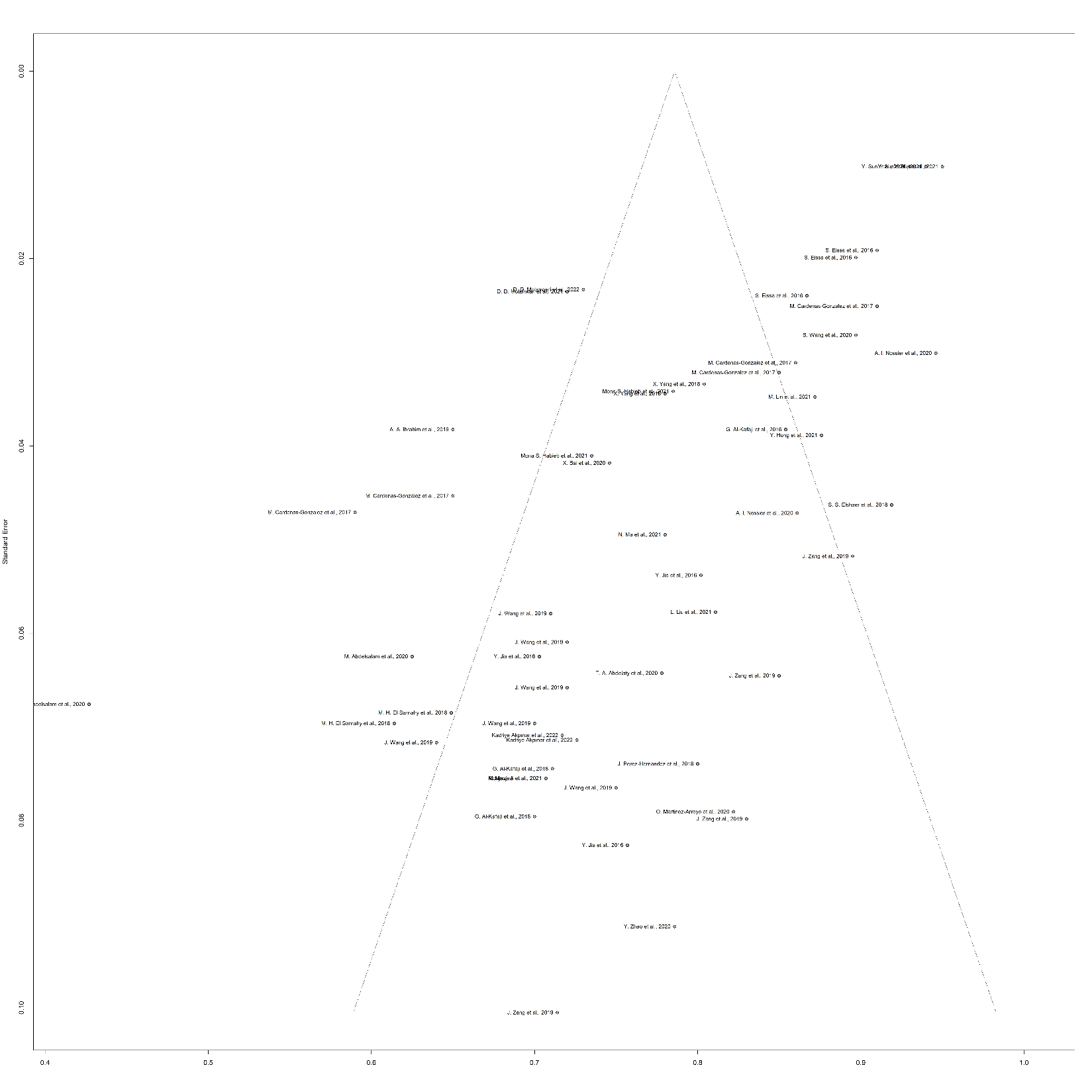


p=<0.001, Egger’s test: There was publication bias

### Supplementary Figure S9. The publication bias for DN vs. healthy control groups’ studies.


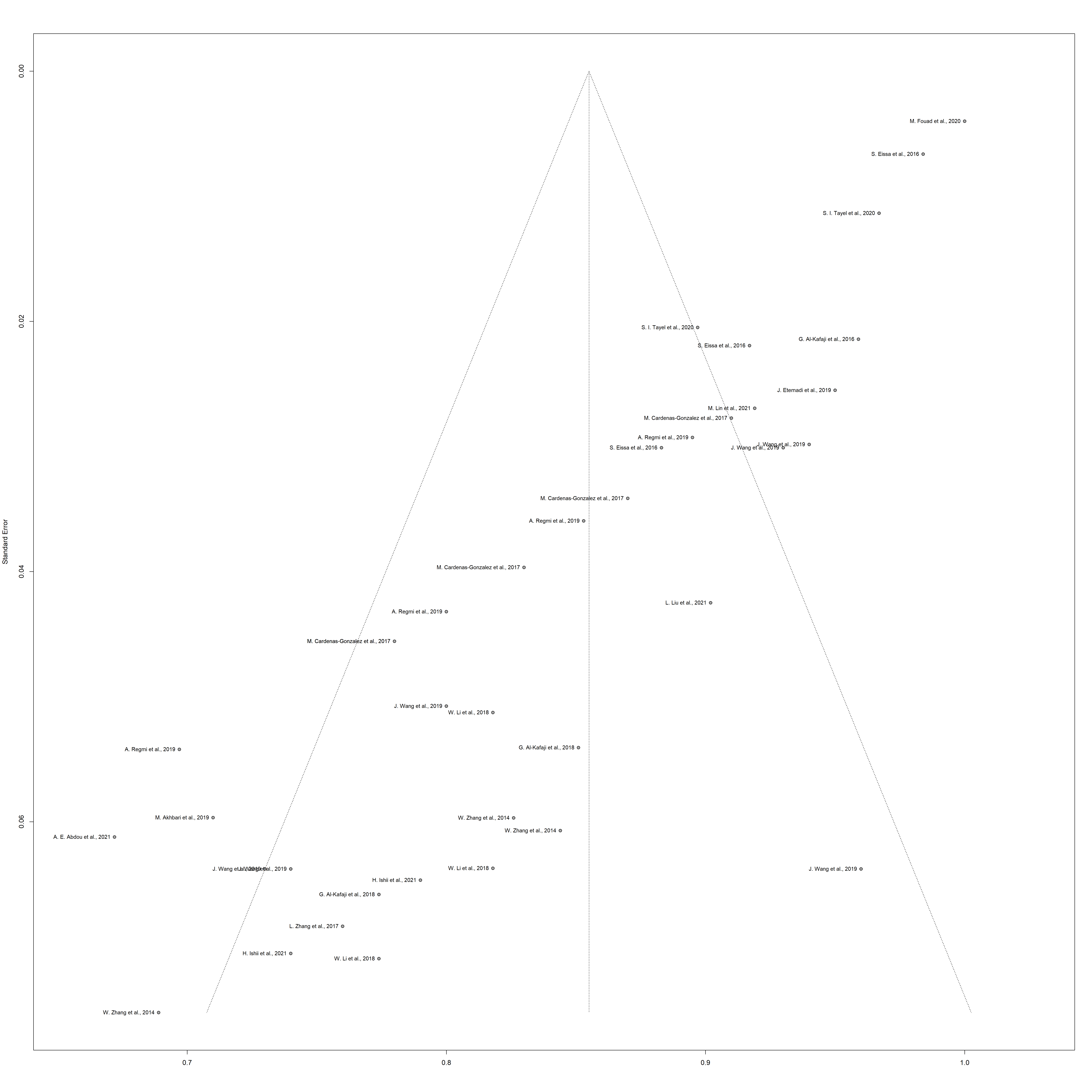


p=<0.001, Egger’s test: There was publication bias

### Supplementary Figure S10. The publication bias for LN vs. SLE studies.

**
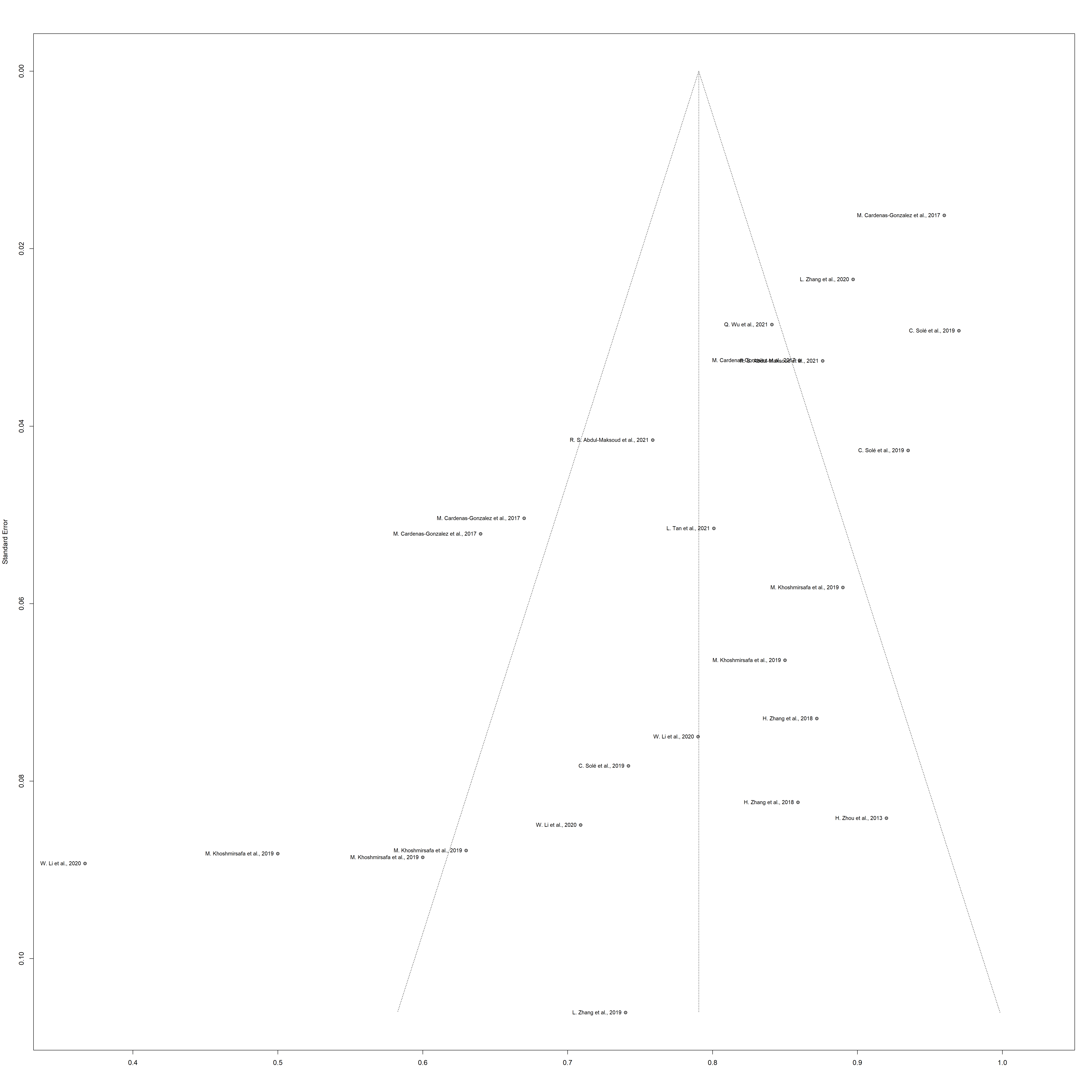
**

p=0.09, Egger’s test: There was no publication bias

### Supplementary Figure S11. The publication bias for LN vs. healthy control studies.

**
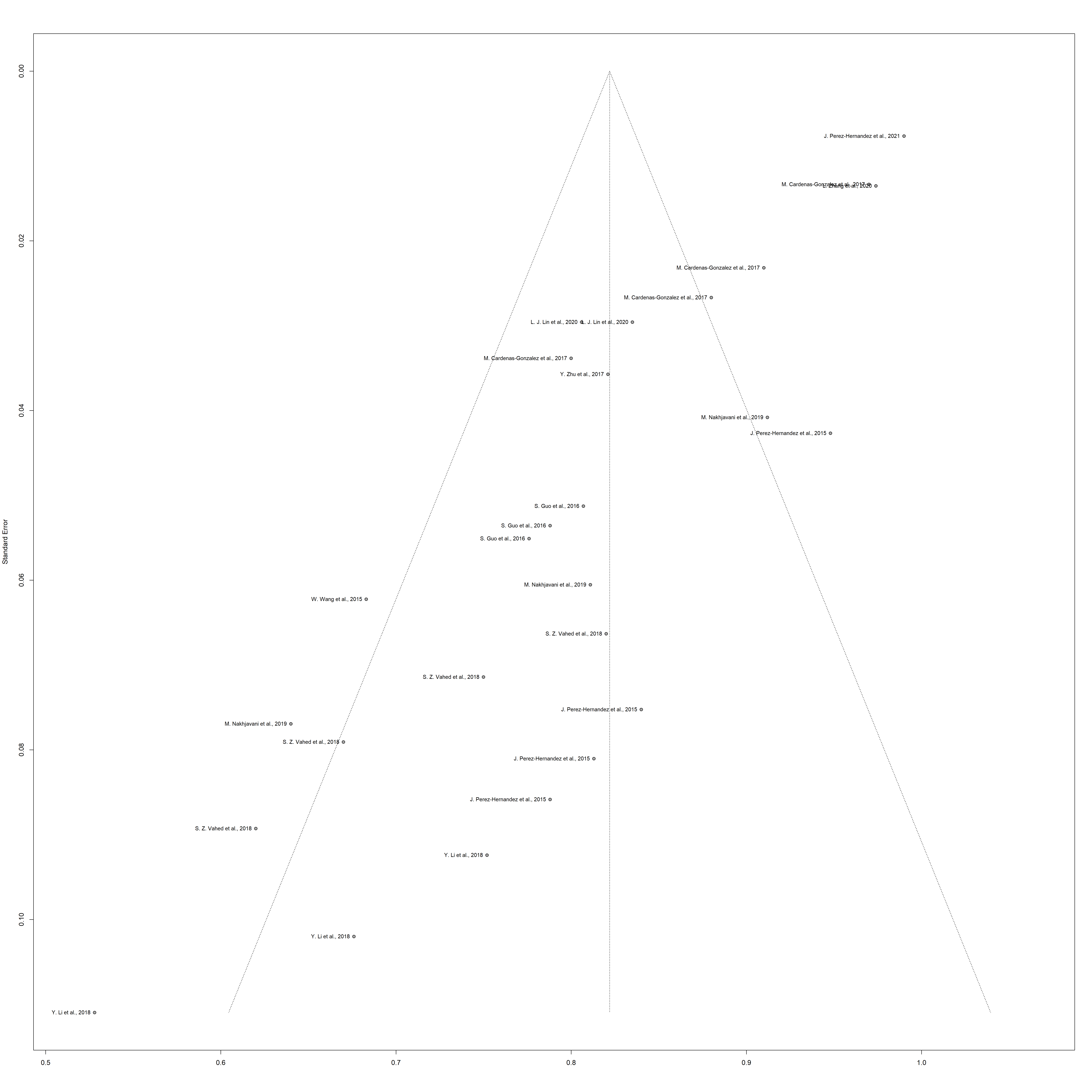
**

p=<0.001, Egger’s test: There was publication bias

### Supplementary Figure S12. The publication bias for IgAN vs. chronic disease groups’ studies.

**
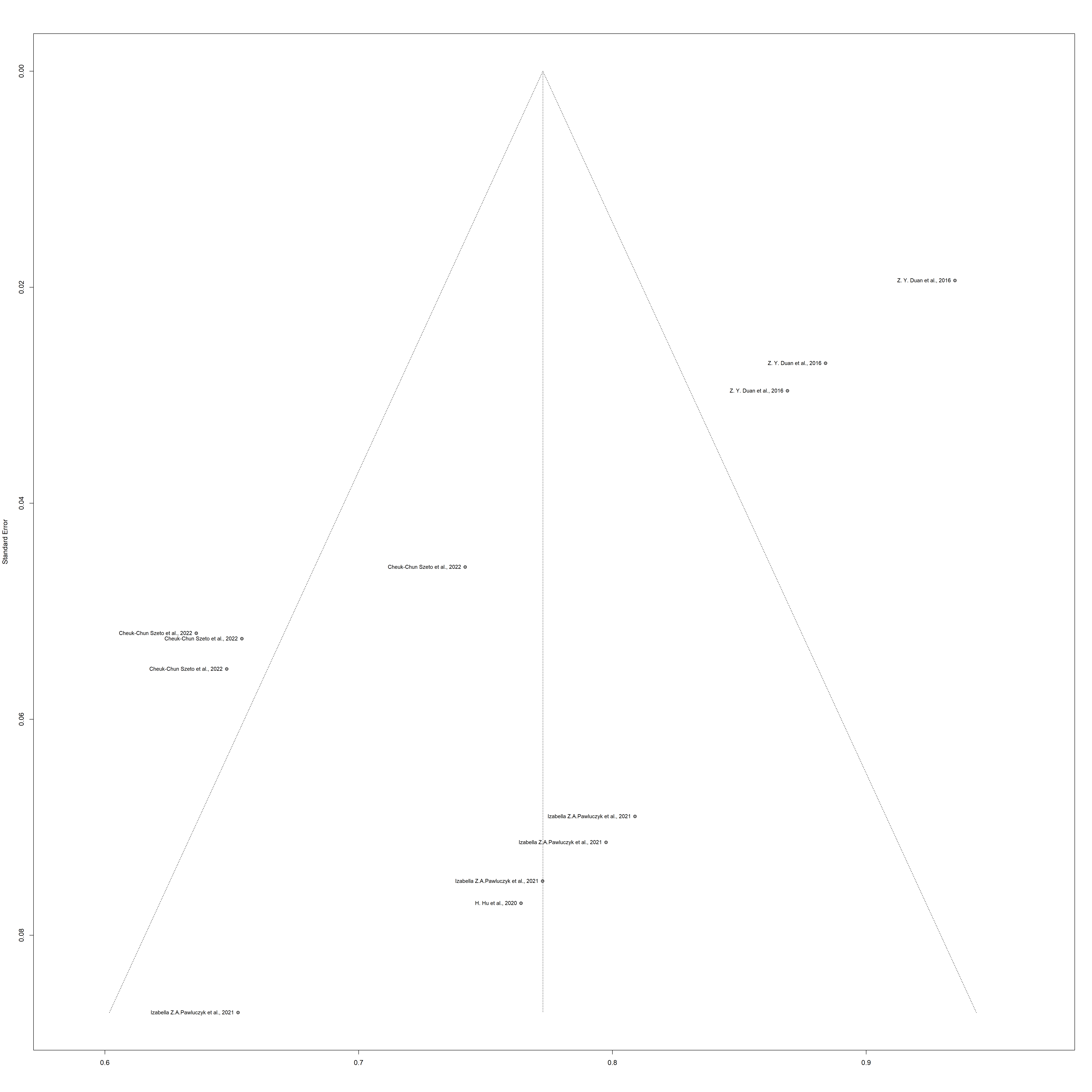
**

### Supplementary Figure S13. The publication bias for IgAN vs. healthy control groups’ studies.

**
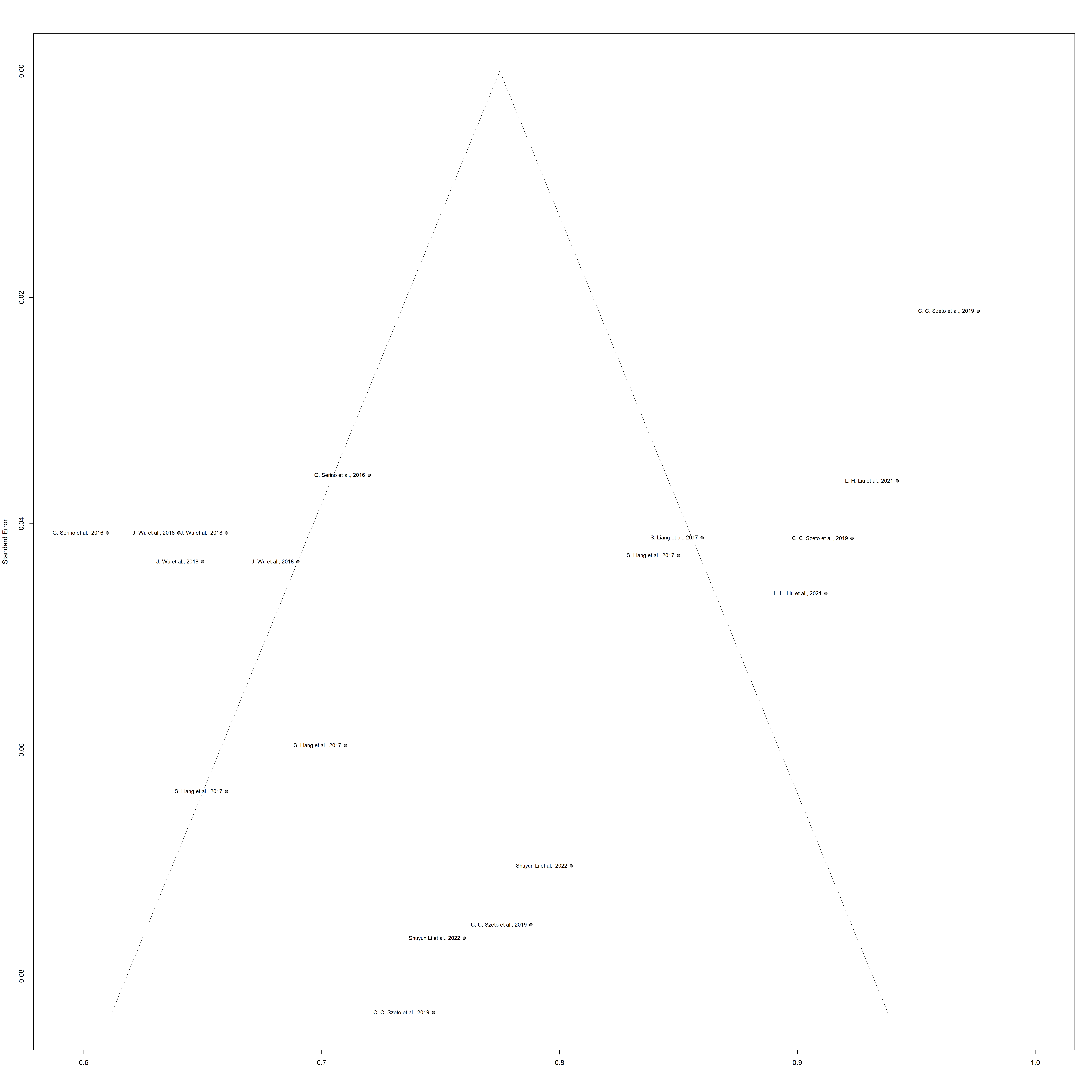
**

### Supplementary Figure S14. The publication bias for MN vs. healthy control groups’s studies.

**
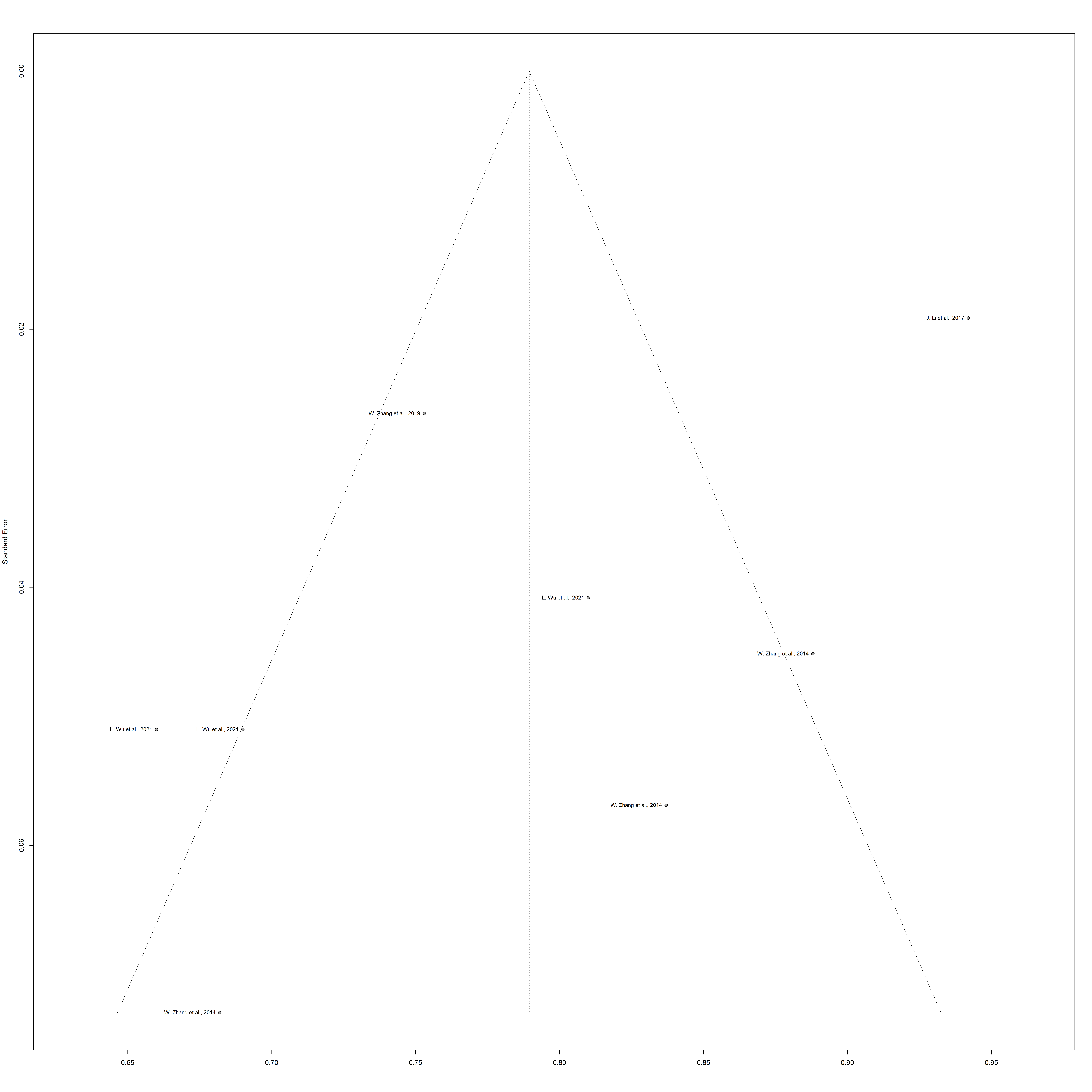
**

### Supplementary Figure S15. The publication bias for FSGS vs. chronic disease groups’ studies.

**
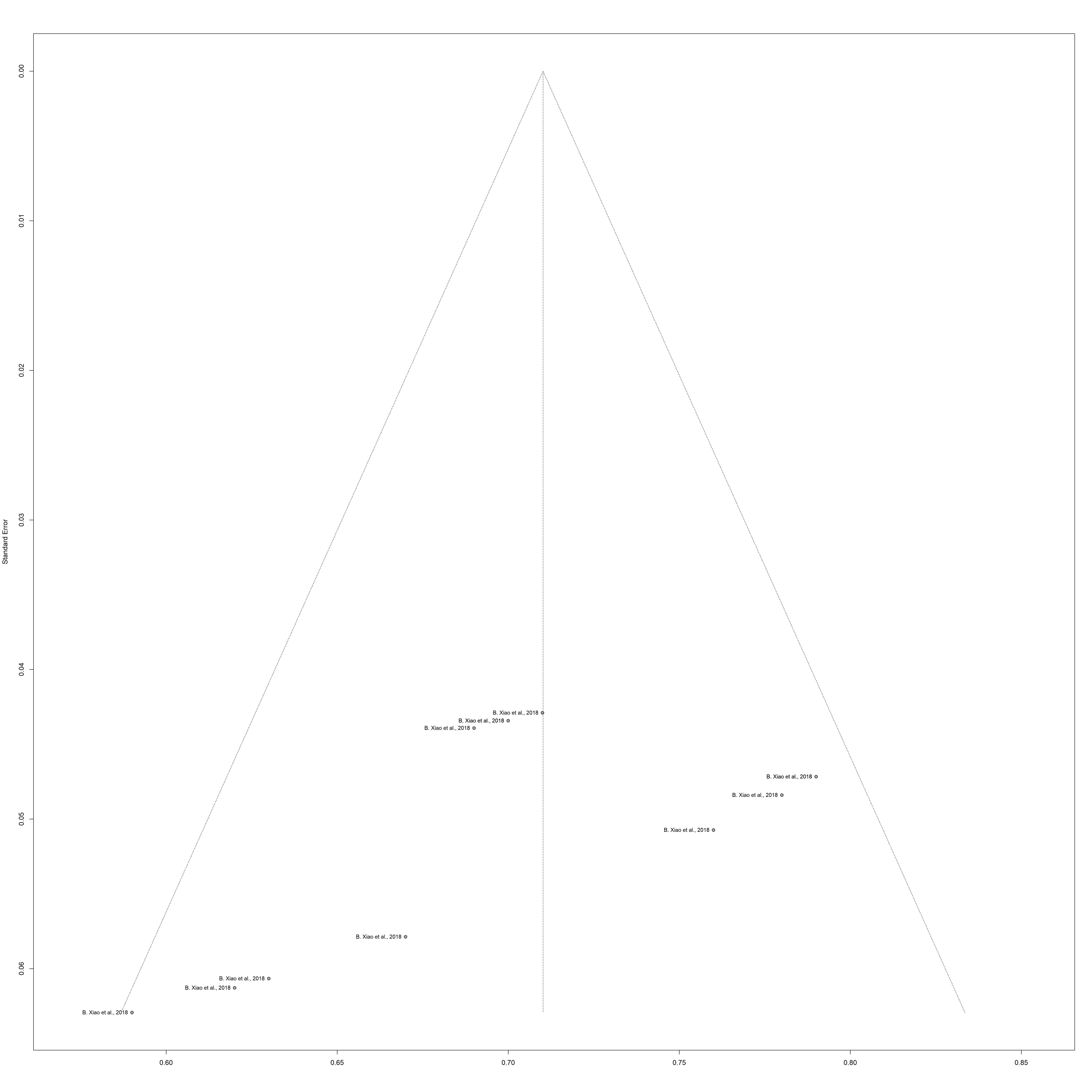
**

### Supplementary Figure S16. The publication bias for FSGS vs. healthy control groups’ studies.

**
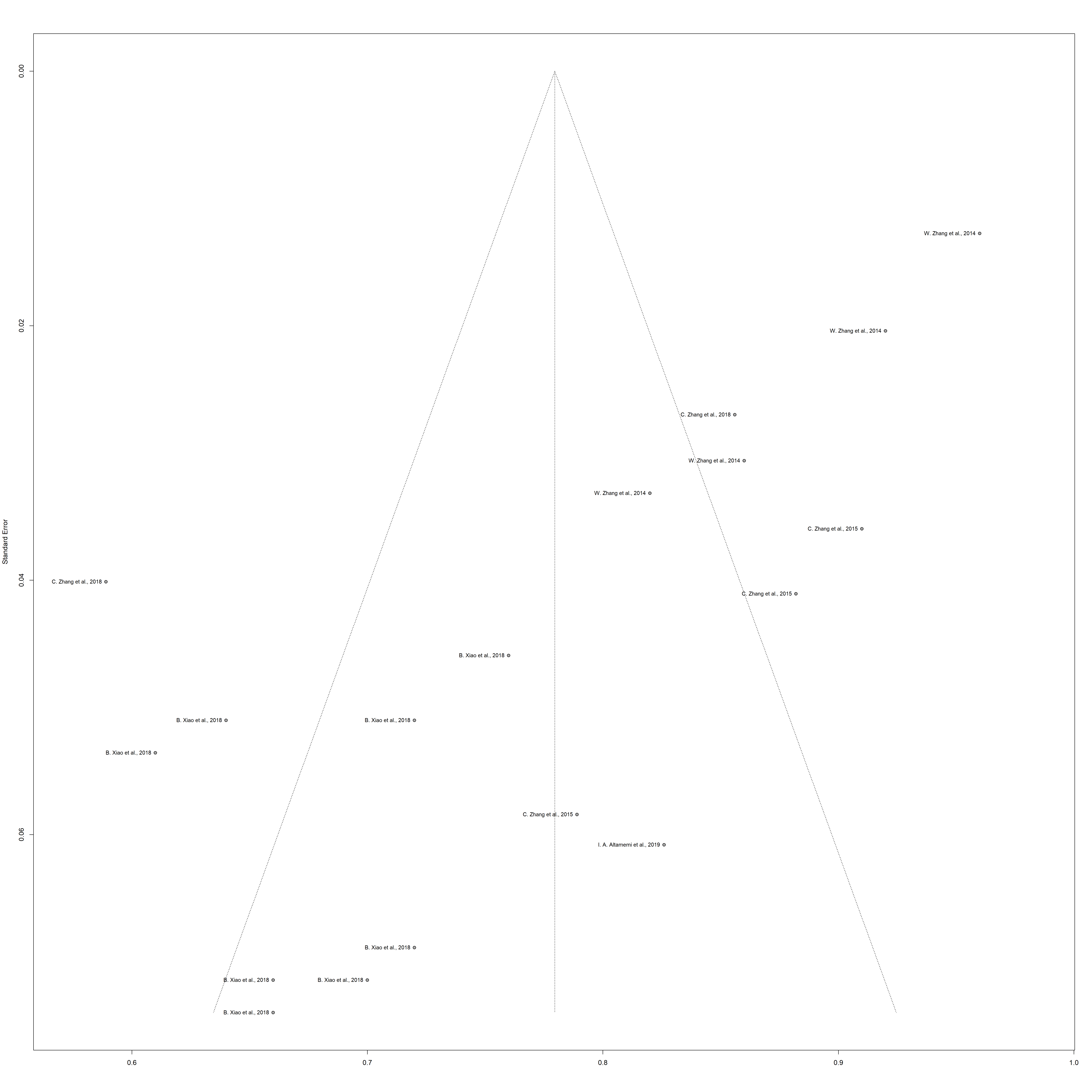
**

### Supplementary Figure S17. Risk of bias assessment by QUADAS-2

**
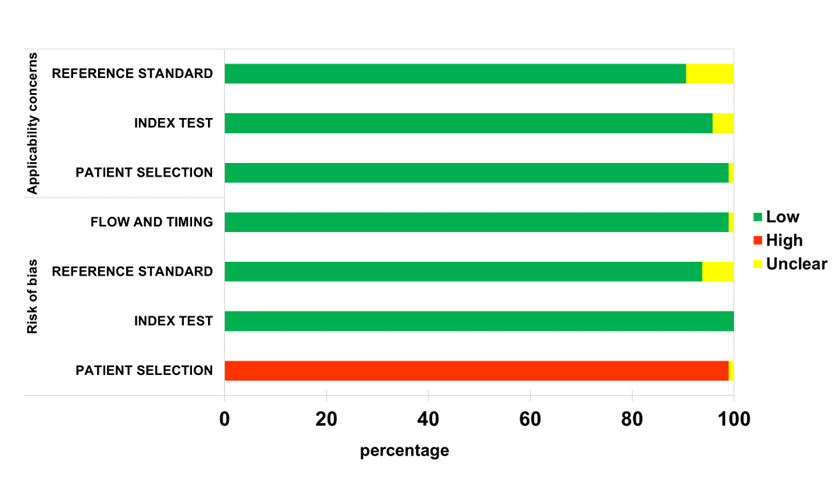
**

Legend: The risk of bias assessment results by QUADAS-2 tools are shown. The color indicates risks; green is low, red is high, and yellow is unclear.

### Supplementary Figure S18. Univariate analysis of AUC value of single miRNAs stratified by sample types in CKD patients compared to healthy controls.


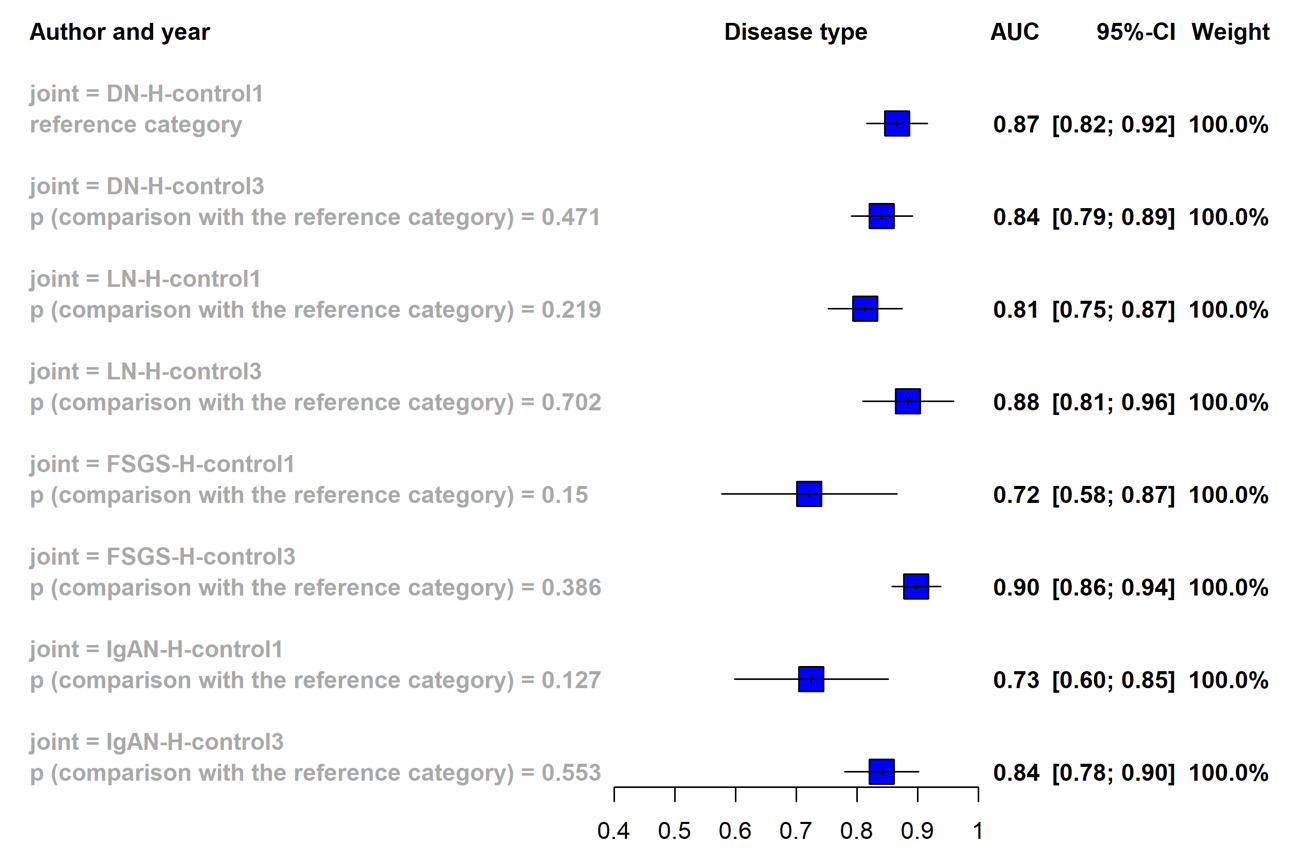


Legend: The overall AUC values of single miRNAs in CKD are shown by different sample types; 1 – blood, 3 – urine and, control types; A. healthy and B. chronic disease groups -abbreviation; H – healthy, D – diseased.

### Supplementary Figure S19. Univariate analysis of single miRNA AUC values in diabetic nephropathy with healthy controls (with sample type stratification)


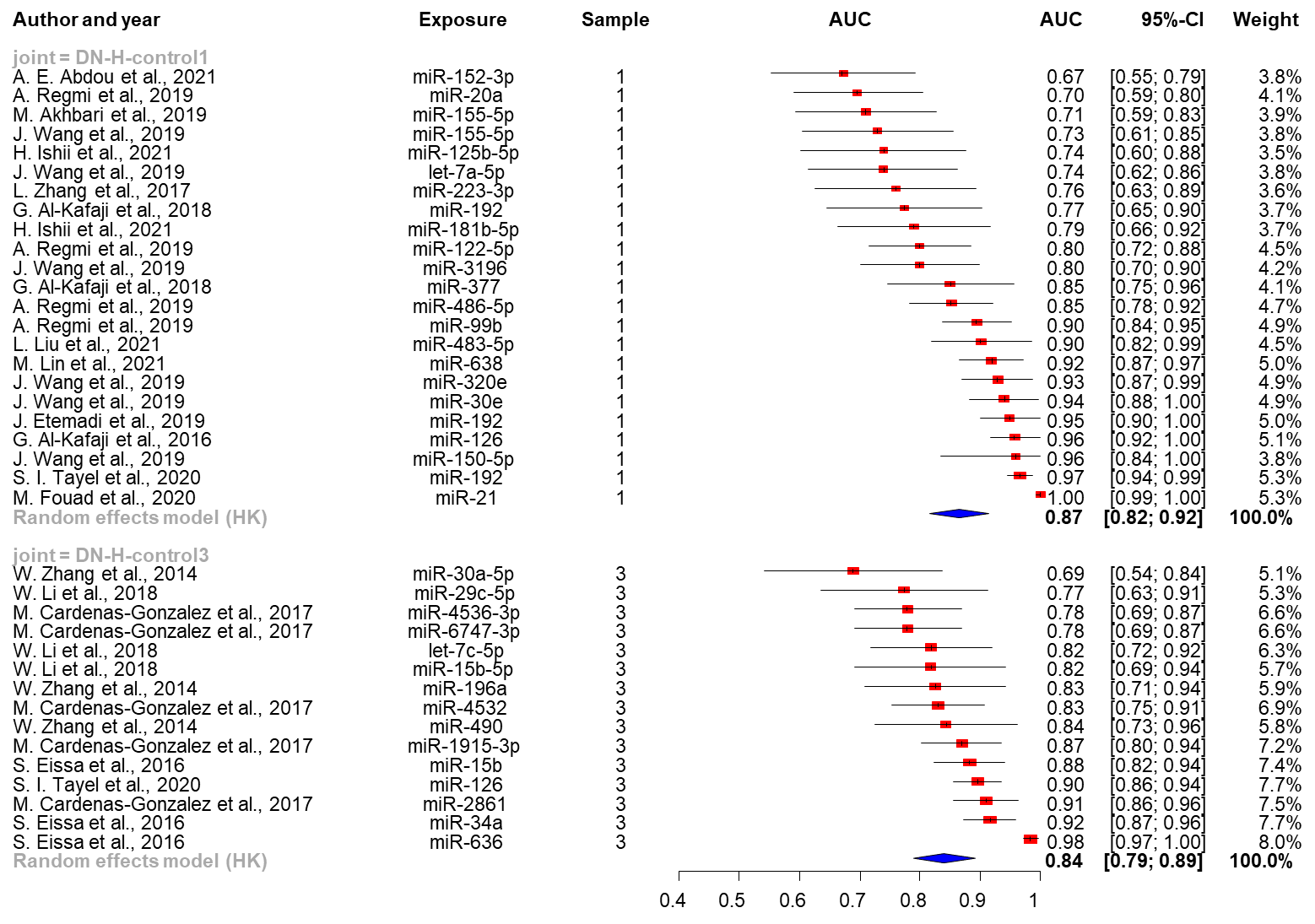


Legend: The overall single miRNA AUC values in diabetic nephropathy (DN) with healthy controls are shown. Sample types stratify results; blood samples are represented by 1, and urines are 3.

### Supplementary Figure S20. Univariate analysis of single miRNA AUC values in lupus nephritis with healthy controls (with sample type stratification)


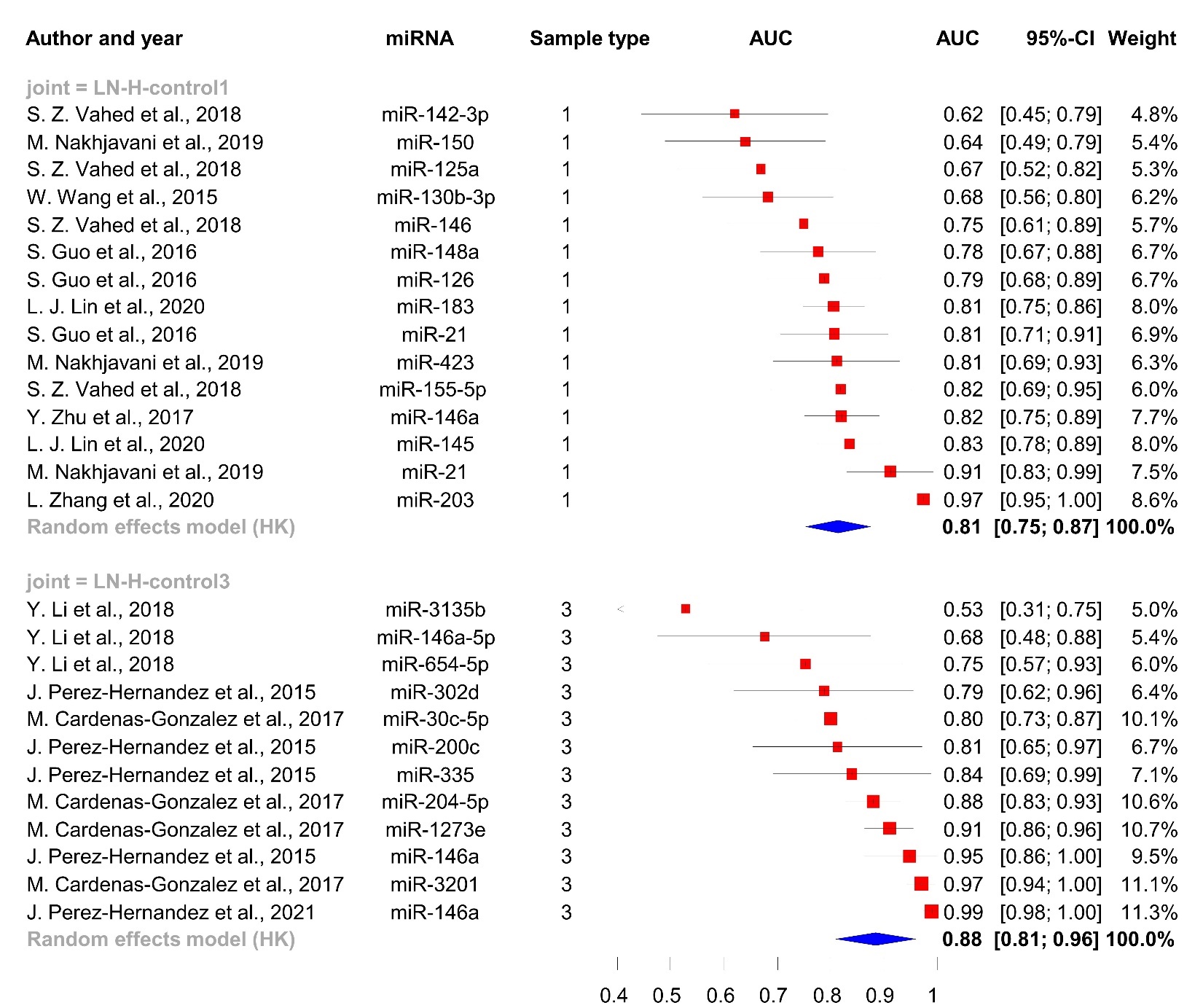


Legend: The overall single miRNA AUC values in lupus nephritis (LN) with healthy controls are shown. Sample types stratify results; blood samples are represented by 1, and urines are 3.

### Supplementary Figure S21. Univariate analysis of single miRNA AUC values in focal segmental glomerulosclerosis with healthy controls (with sample type stratification)


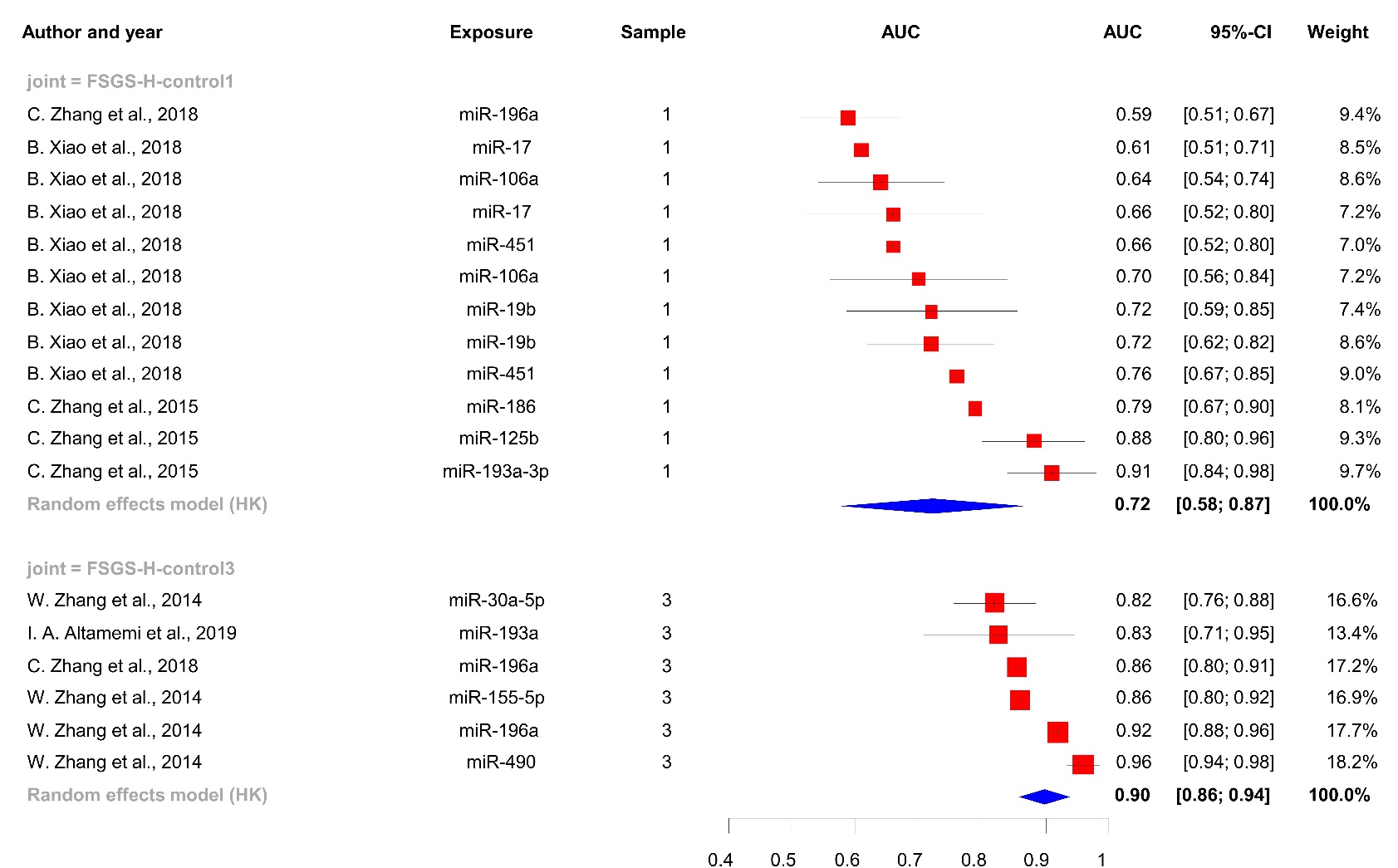


**joint = FSGS-H-control1**

**joint = FSGS-H-control3**

**Random effects model (HK)**

**Random effects model (HK)**

C. Zhang et al., 2018

B. Xiao et al., 2018

B. Xiao et al., 2018

B. Xiao et al., 2018

B. Xiao et al., 2018

B. Xiao et al., 2018

B. Xiao et al., 2018

B. Xiao et al., 2018

B. Xiao et al., 2018

C. Zhang et al., 2015

C. Zhang et al., 2015

C. Zhang et al., 2015

W. Zhang et al., 2014

I. A. Altamemi et al., 2019

C. Zhang et al., 2018

W. Zhang et al., 2014

W. Zhang et al., 2014

W. Zhang et al., 2014

miR-196a

miR-17

miR-106a

miR-17

miR-451

miR-106a

miR-19b

miR-19b

miR-451

miR-186

miR-125b

miR-193a-3p

miR-30a-5p

miR-193a

miR-196a

miR-155-5p

miR-196a

miR-490

1

1

1

1

1

1

1

1

1

1

1

1

3

3

3

3

3

3

0.4

0.5

0.6

0.7

0.8

0.9

1

**0.72**

**0.90**

0.59

0.61

0.64

0.66

0.66

0.70

0.72

0.72

0.76

0.79

0.88

0.91

0.82

0.83

0.86

0.86

0.92

0.96

**[0.58; 0.87]**

**[0.86; 0.94]**

[0.51; 0.67]

[0.51; 0.71]

[0.54; 0.74]

[0.52; 0.80]

[0.52; 0.80]

[0.56; 0.84]

[0.59; 0.85]

[0.62; 0.82]

[0.67; 0.85]

[0.67; 0.90]

[0.80; 0.96]

[0.84; 0.98]

[0.76; 0.88]

[0.71; 0.95]

[0.80; 0.91]

[0.80; 0.92]

[0.88; 0.96]

[0.94; 0.98]

**Author and year**

**Exposure**

**Sample**

**AUC**

**AUC**

**95%-CI**

**Weight**

**100.0%**

**100.0%**

9.4%

8.5%

8.6%

7.2%

7.0%

7.2%

7.4%

8.6%

9.0%

8.1%

9.3%

9.7%

16.6%

13.4%

17.2%

16.9%

17.7%

18.2%

Legend: The overall single miRNA AUC values in focal segmental glomerulosclerosis (FSGS) with healthy controls are shown. Sample types stratify results; blood samples are represented by 1, and urines are 3.

**Supplementary Figure S22. Univariate analysis of single miRNA AUC values in IgA nephropathy with healthy controls (with sample type stratification)**


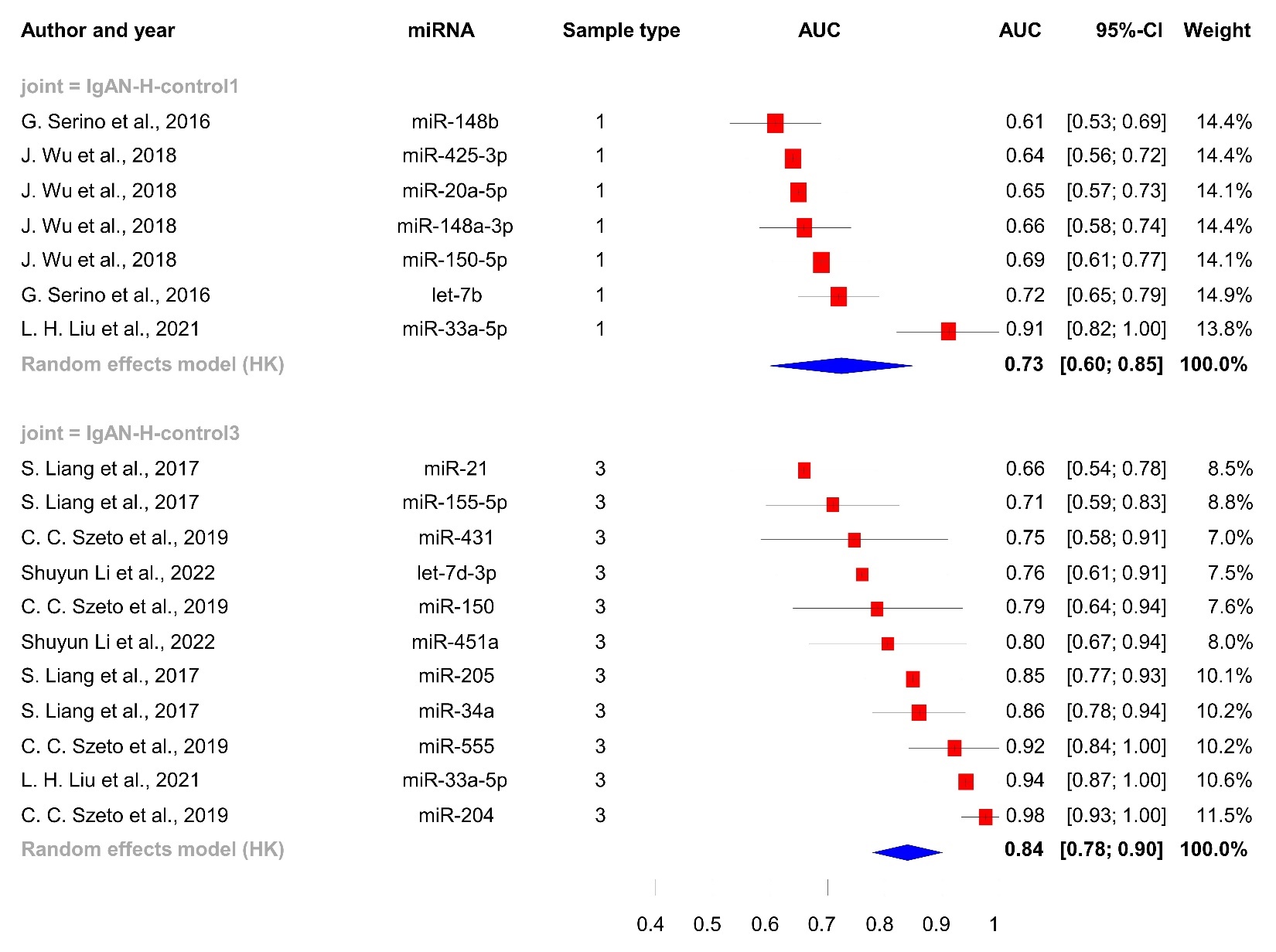


Legend: The overall single miRNA AUC values in IgA nephropathy (IgAN) with healthy controls are shown. Sample types stratify results; blood samples are represented by 1, and urines are 3.

### Supplementary Figure S23. Univariate analysis of single miRNA AUC values in membranous nephropathy with healthy controls (with sample type stratification)


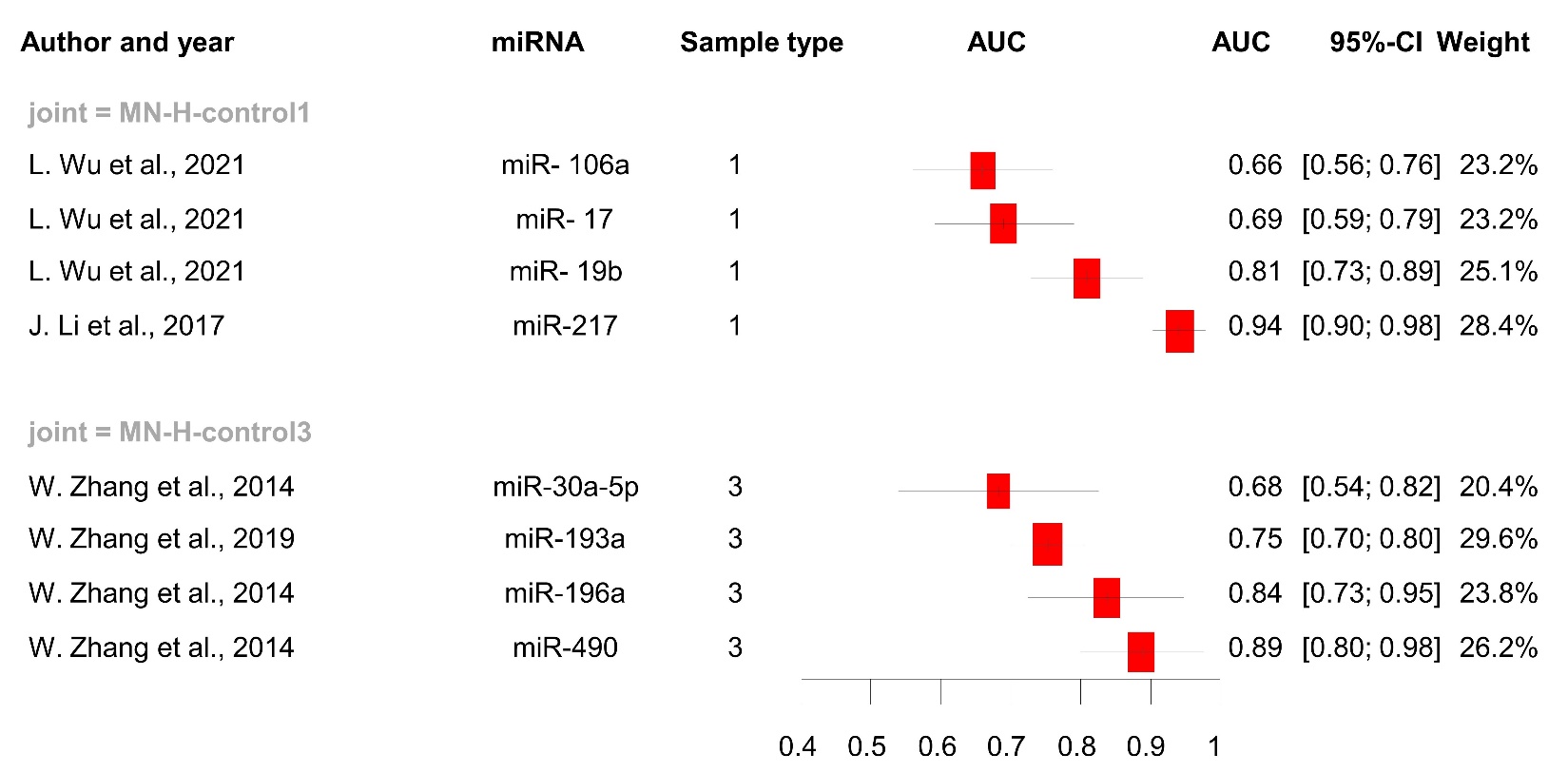


Legend: The overall single miRNA AUC values in membranous nephropathy (MN) with healthy controls are shown. Sample types stratify results; blood samples are represented by 1, and urines are 3.

### Supplementary Figure S24. The pooled AUC values of miR-21 in CKD comparison with A. Healthy controls and, B. Chronic disease groups.

**A.**


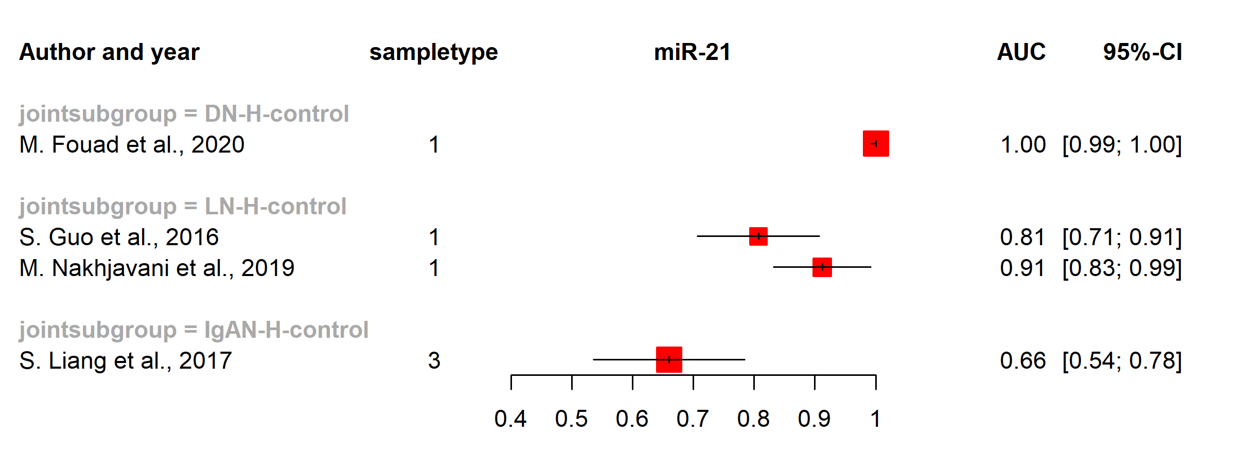


**B.**


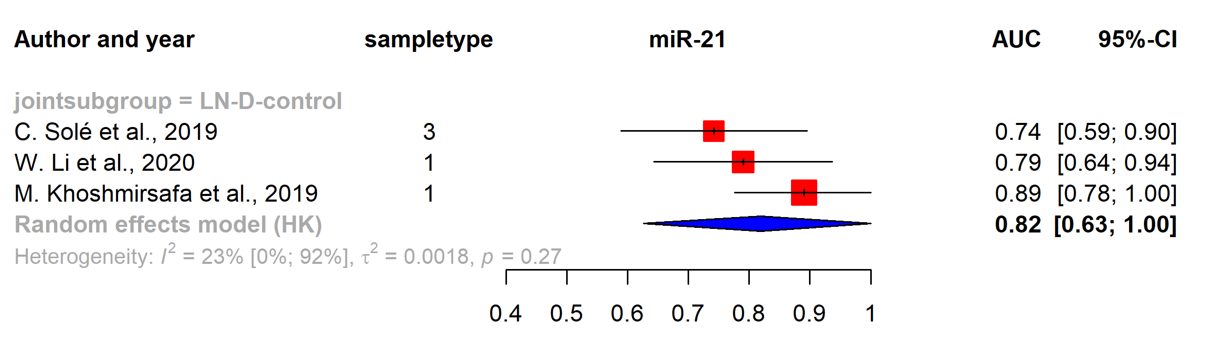


### Supplementary Table S5. Risk of bias assessment, QUADAS-2

| **Study** | **Risk of bias** | | | | **Applicability concerns** | | |
| --- | --- | --- | --- | --- | --- | --- | --- |
|  | **Patient selection** | **Index test** | **Reference standard** | **Flow and timing** | **Patient selection** | **Index test** | **Reference standard** |
| T. A. Abdelaty, 2020 |  |  |  |  |  |  |  |
| M. Abdelsalam, 2020 |  |  |  |  |  |  |  |
| R. S. Abdul-Maksoud, 2021 |  |  |  |  |  |  |  |
| A. E. Abdou, 2021 |  |  |  |  |  |  |  |
| M. Akhbari, 2019 |  |  |  |  |  |  |  |
| G. Al-Kafaji, 2016 |  |  |  |  |  |  |  |
| I. A. Altamemi, 2019 |  |  |  |  |  |  |  |
| M. Ardalan, 2020 |  |  |  |  |  |  |  |
| X. Bai, 2020 |  |  |  |  |  |  |  |
| N. R. Bayomy, 2021 |  |  |  |  |  |  |  |
| C. Beltrami, 2018 |  |  |  |  |  |  |  |
| M. Cardenas-Gonzalez, 2017 |  |  |  |  |  |  |  |
| T. Chen, 2019 |  |  |  |  |  |  |  |
| L. Chun-yan, 2018 |  |  |  |  |  |  |  |
| Z. Y. Duan, 2016 |  |  |  |  |  |  |  |
| S. Eissa, 2016 |  |  |  |  |  |  |  |
| M. H. El-Samahy, 2018 |  |  |  |  |  |  |  |
| S. S. Elshaer, 2018 |  |  |  |  |  |  |  |
| J. Etemadi, 2019 |  |  |  |  |  |  |  |
| D. Feng, 2020 |  |  |  |  |  |  |  |
| M. Fouad, 2020 |  |  |  |  |  |  |  |
| S. Guo, 2016 |  |  |  |  |  |  |  |
| S. M. Hejazian, 2020 |  |  |  |  |  |  |  |
| Y. Hong, 2021 |  |  |  |  |  |  |  |
| H. Hu, 2020 |  |  |  |  |  |  |  |
| C. Huang, 2020 |  |  |  |  |  |  |  |
| P. Huang, 2021 |  |  |  |  |  |  |  |
| Y. Q. Huang, 2018 |  |  |  |  |  |  |  |
| A. A. Ibrahim, 2019 |  |  |  |  |  |  |  |
| H. Ishii, 2021 |  |  |  |  |  |  |  |
| Y. Jia, 2016 |  |  |  |  |  |  |  |
| M. Khoshmirsafa, 2019 |  |  |  |  |  |  |  |
| H. Li, 2021 |  |  |  |  |  |  |  |
| J. Li, 2017 |  |  |  |  |  |  |  |
| W. Li, 2020 |  |  |  |  |  |  |  |
| W. Li, 2018 |  |  |  |  |  |  |  |
| S. Liang, 2017 |  |  |  |  |  |  |  |
| L. J. Lin, 2020 |  |  |  |  |  |  |  |
| M. Lin, 2021 |  |  |  |  |  |  |  |
| L. Liu, 2021 |  |  |  |  |  |  |  |
| L. H. Liu, 2021 |  |  |  |  |  |  |  |
| Y. Luo, 2013 |  |  |  |  |  |  |  |
| C. Y. Lv, 2018 |  |  |  |  |  |  |  |
| L. L. Lv, 2013 |  |  |  |  |  |  |  |
| N. Ma, 2021 |  |  |  |  |  |  |  |
| O. Martinez-Arroyo, 2020 |  |  |  |  |  |  |  |
| A. Monjezi, 2021 |  |  |  |  |  |  |  |
| D. D. Motshwari, 2021 |  |  |  |  |  |  |  |
| M. Nakhjavani, 2019 |  |  |  |  |  |  |  |
| E. Navarro-Quiroz, 2016 |  |  |  |  |  |  |  |
| A. I. Nossier, 2020 |  |  |  |  |  |  |  |
| J. Perez-Hernandez, 2015 |  |  |  |  |  |  |  |
| P. Prabu, 2019 |  |  |  |  |  |  |  |
| A. Regmi, 2019 |  |  |  |  |  |  |  |
| G. Serino, 2016 |  |  |  |  |  |  |  |
| C. Solé, 2019 |  |  |  |  |  |  |  |
| W. Sui, 2014 |  |  |  |  |  |  |  |
| Y. Sun, 2021 |  |  |  |  |  |  |  |
| C. C. Szeto, 2019 |  |  |  |  |  |  |  |
| L. Tan, 2021 |  |  |  |  |  |  |  |
| S. I. Tayel, 2020 |  |  |  |  |  |  |  |
| S. Z. Vahed, 2018 |  |  |  |  |  |  |  |
| J. Wang, 2019 |  |  |  |  |  |  |  |
| L. Wang, 2020 |  |  |  |  |  |  |  |
| S. Wang, 2020 |  |  |  |  |  |  |  |
| W. Wang, 2015 |  |  |  |  |  |  |  |
| Z. G. Wang, 2020 |  |  |  |  |  |  |  |
| J. Wu, 2018 |  |  |  |  |  |  |  |
| L. Wu, 2021 |  |  |  |  |  |  |  |
| Q. Wu, 2021 |  |  |  |  |  |  |  |
| B. Xiao, 2018 |  |  |  |  |  |  |  |
| X. Yang, 2018 |  |  |  |  |  |  |  |
| M. P. Yavropoulou, 2020 |  |  |  |  |  |  |  |
| C. Zhang, 2018 |  |  |  |  |  |  |  |
| H. Zhang, 2018 |  |  |  |  |  |  |  |
| L. Zhang, 2017 |  |  |  |  |  |  |  |
| W. Zhang, 2019 |  |  |  |  |  |  |  |
| Y. Zhao, 2020 |  |  |  |  |  |  |  |
| H. Zhou, 2013 |  |  |  |  |  |  |  |
| Y. Zhu, 2017 |  |  |  |  |  |  |  |
| Shuyun Li, 2022 |  |  |  |  |  |  |  |
| Mona S. Habieb, 2021 |  |  |  |  |  |  |  |
| Cheuk-Chun Szeto, 2022 |  |  |  |  |  |  |  |
| Monjezi, A, 2021 |  |  |  |  |  |  |  |
| Izabella Z.A.Pawluczyk, 2021 |  |  |  |  |  |  |  |
| Kadriye Akpınar, 2022 |  |  |  |  |  |  |  |

| low | unclear | high |
| --- | --- | --- |

# REFERENCES

1. Freeman, S.C., et al., *Development of an interactive web-based tool to conduct and interrogate meta-analysis of diagnostic test accuracy studies: MetaDTA.* BMC Med Res Methodol, 2019. **19**(1): p. 81.

2. Hanley, J.A. and B.J. McNeil, *The meaning and use of the area under a receiver operating characteristic (ROC) curve.* Radiology, 1982. **143**(1): p. 29-36.

3. Pustejovsky, J.E. and E. Tipton, *Meta-analysis with Robust Variance Estimation: Expanding the Range of Working Models.* Prev Sci, 2022. **23**(3): p. 425-438.

4. Rutter, C.M. and C.A. Gatsonis, *A hierarchical regression approach to meta-analysis of diagnostic test accuracy evaluations.* Stat Med, 2001. **20**(19): p. 2865-84.

5. Reitsma, J.B., et al., *Bivariate analysis of sensitivity and specificity produces informative summary measures in diagnostic reviews.* J Clin Epidemiol, 2005. **58**(10): p. 982-90.

6. Chu, H. and S.R. Cole, *Bivariate meta-analysis of sensitivity and specificity with sparse data: a generalized linear mixed model approach.* J Clin Epidemiol, 2006. **59**(12): p. 1331-2; author reply 1332-3.

7. Harbord, R.M., et al., *A unification of models for meta-analysis of diagnostic accuracy studies.* Biostatistics, 2007. **8**(2): p. 239-51.

8. Burke, D.L., et al., *Guidance for deriving and presenting percentage study weights in meta-analysis of test accuracy studies.* Res Synth Methods, 2018. **9**(2): p. 163-178.
